# GPNMB disrupts SNARE complex assembly to maintain bacterial proliferation within macrophages

**DOI:** 10.1038/s41423-025-01272-z

**Published:** 2025-03-04

**Authors:** Zhenzhen Yan, Jinghong Han, Zihao Mi, Zhenzhen Wang, Yixuan Fu, Chuan Wang, Ningning Dang, Hong Liu, Furen Zhang

**Affiliations:** 1 Hospital for Skin Diseases, Shandong First Medical University, Jinan, Shandong China; 2 Shandong Provincial Institute of Dermatology and Venereology, Shandong Academy of Medical Sciences, Jinan, Shandong China; 3 Department of Dermatology, Shandong Provincial Hospital Affiliated with Shandong First Medical University, Jinan, Shandong China; 4 School of Public Health, Shandong First Medical University & Shandong Academy of Medical Sciences, Jinan, Shandong China

**Keywords:** Intracellular bacterial infection, Leprosy, Xenophagy, SNARE complex, GPNMB, SNAP29, Autophagy, Antimicrobial responses, Bacterial infection

## Abstract

Xenophagy plays a crucial role in restraining the growth of intracellular bacteria in macrophages. However, the machinery governing autophagosome‒lysosome fusion during bacterial infection remains incompletely understood. Here, we utilize leprosy, an ideal model for exploring the interactions between host defense mechanisms and bacterial infection. We highlight the glycoprotein nonmetastatic melanoma protein B (GPNMB), which is highly expressed in macrophages from lepromatous leprosy (L-Lep) patients and interferes with xenophagy during bacterial infection. Upon infection, GPNMB interacts with autophagosomal-localized STX17, leading to a reduced N-glycosylation level at N296 of GPNMB. This modification promotes the degradation of SNAP29, thus preventing the assembly of the STX17-SNAP29-VAMP8 SNARE complex. Consequently, the fusion of autophagosomes with lysosomes is disrupted, resulting in inhibited cellular autophagic flux. In addition to *Mycobacterium leprae*, GPNMB deficiency impairs the proliferation of various intracellular bacteria in human macrophages, suggesting a universal role of GPNMB in intracellular bacterial infection. Furthermore, compared with their counterparts, *Gpnmb*^fl/fl^ Lyz2-Cre mice presented decreased *Mycobacterium marinum* amplification. Overall, our study reveals a previously unrecognized role of GPNMB in host antibacterial defense and provides insights into its regulatory mechanism in SNARE complex assembly.

## Introduction

Intracellular bacterial pathogens cause a wide range of diseases and significantly contribute to chronic, persistent, and latent infection, posing a serious threat to public health [1]. These pathogens evade host immune surveillance and replicate inside host cells, primarily macrophages [2]. Upon bacterial infection, multiple cellular signaling pathways in macrophages are activated and subsequently initiate specific immune responses to eliminate invading pathogens. Xenophagy is an important innate immune mechanism employed by macrophages to inhibit the intracellular survival of these pathogens [3]. During xenophagy, autophagosomes sequester intracellular bacteria either in the cytosol or within damaged vacuoles and then deliver bacterial contents to lysosomes for degradation [4]. Exploring the mechanisms of autophagosome‒lysosome fusion during bacterial infection is crucial for understanding the immune evasion strategies of intracellular bacteria.

The soluble N-ethylmaleimide-sensitive factor attachment protein receptor (SNARE) complex is a key regulator of the fusion of autophagosomes with late endosomes/lysosomes. The SNARE complex is composed of autophagosomal localized syntaxin 17 (STX17), synaptosome associated protein 29 (SNAP29), and late endosomal/lysosomal localized vesicle associated membrane protein 8 (VAMP8) or, alternatively, autophagosomal localized YKT6 v-SNARE homolog (YKT6), SNAP29 and late endosomal/lysosomal localized syntaxin 7 (STX7). Some reports have shown that these two sets of SNARE complexes have partially redundant functions [5–7], whereas a recent report has demonstrated that STX17 and YKT6 operate in the same linear pathway [8]. Researchers have predicted that different SNARE complexes may facilitate the fusion of autophagic vacuoles with different sets of late endosomes/lysosomes [5, 8]. However, the specific SNARE complex responsible for the maturation of autophagosomes in xenophagy upon bacterial infection has not been clearly defined. Additionally, the precise mechanisms regulating SNARE complex assembly need to be further explored.

*Mycobacterium leprae* (*M. leprae*), the etiologic agent of leprosy, was the first human intracellular pathogen discovered [9]. Upon *M. leprae* infection, leprosy manifests as a spectrum of skin diseases, ranging from a few well-delimited lesions with undetectable bacilli (tuberculoid leprosy, T-Lep) to multiple lesions with high bacillary loads (lepromatous leprosy, L-Lep) [10]. Clinical manifestations correlate with the host immune response to the pathogen [11, 12]. Previous studies have shown the presence of *M. leprae*-containing double-membrane vacuoles in mouse macrophages [13] and in macrophages isolated from the skin lesions of leprosy patients [14], suggesting the possible involvement of xenophagy in the immunomodulatory response to this disease. Moreover, patients with the L-Lep form have been found to exhibit lower levels of xenophagy than those with the T-Lep form [15]. However, the mechanism through which *M. leprae* evades host killing through disruption of xenophagy in L-Lep needs further exploration.

Although *M. leprae* has been known for more than 100 years, it currently cannot be cultured in vitro [11]. Additionally, effective animal models for performing leprosy immunological studies are lacking [11, 16]. A previous study [17] indicated that *Mycobacterium marinum* (*M. marinum*), the closest genetic relative of the *M. tuberculosis* complex [18], shares many infection features with *M. leprae*, particularly in terms of interactions with macrophages [11]. Like *M. leprae*, *M. marinum* is a macrophage pathogen that causes chronic, systemic disease [18]. Thus, in this study, we constructed in vitro and in vivo infection models by using *M. marinum*.

Glycoprotein nonmetastatic melanoma protein B (GPNMB), a glycosylated type I transmembrane protein, is widely expressed in various tissues, including the skin [19], brain [20], breast [21], muscle [22], and bone [23]. High expression of GPNMB is closely associated with various tumors, such as breast cancer [21], glioblastoma [24], and lung cancer [25], and promotes tumor growth, invasion, and metastasis [25]. Additionally, GPNMB is linked to various pathological conditions, including Parkinson’s disease [20], obesity [26], glaucoma [25], and neuroinflammation [27]. In our previous study [28], we detected higher mRNA levels of *GPNMB* in macrophages from patients with L-Lep than in those from patients with T-Lep, suggesting a close relationship between GPNMB and *M. leprae* survival. However, the role of GPNMB in the host defense against bacterial infection remains unclear. Our findings revealed that GPNMB promoted intracellular bacterial survival by blocking xenophagy in macrophages. Indeed, GPNMB was recruited to microtubule-associated protein 1 light chain 3 (LC3)-containing vacuoles upon *M. leprae* or *M. marinum* infection. During infection, GPNMB bound to autophagosomal-localized STX17, leading to the deglycosylation of GPNMB, which promoted SNAP29 degradation. This process inhibits the assembly of the STX17-SNAP29-VAMP8 SNARE complex, prevents the fusion of autophagosomes with lysosomes and disrupts cellular autophagic flux. Our results reveal the negative regulatory role of GPNMB in the intracellular bacterial immune response and propose that GPNMB is an attractive drug target for combating intracellular bacterial infection.

## Results

### GPNMB overexpression is closely related to a weak immune state in L-Lep

**Fig. 1:**
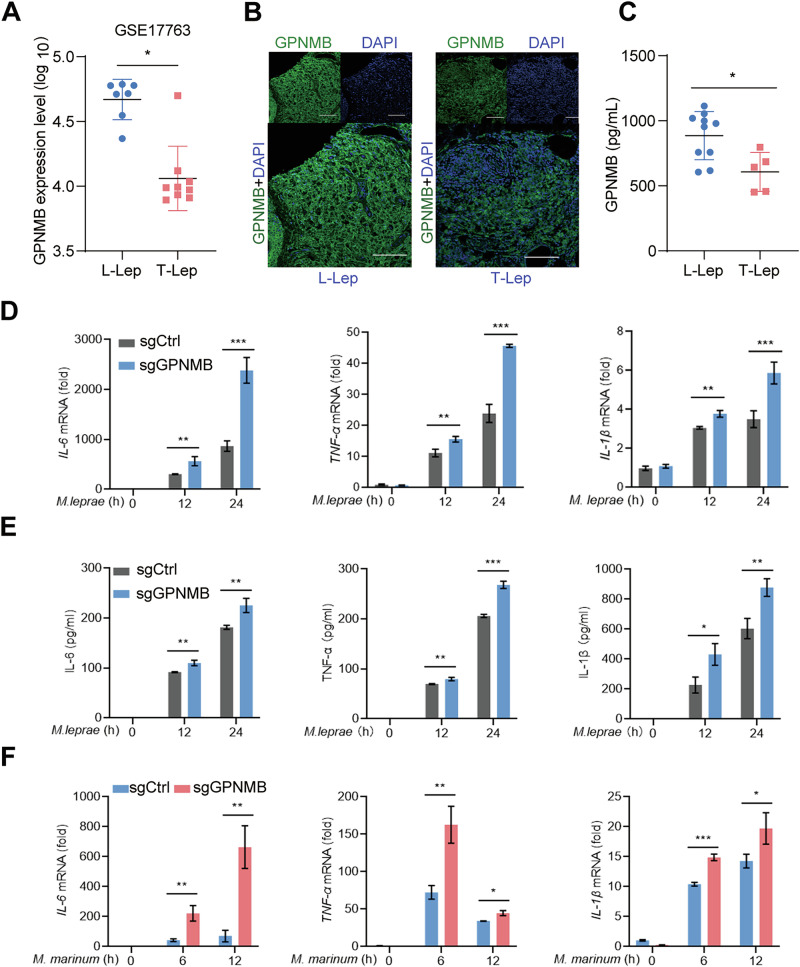
GPNMB deficiency promotes the production of proinflammatory cytokines during bacterial infection. **A** The expression level of GPNMB in the skin of different leprosy patients (GSE17763). **B** mIHC image showing the expression of GPNMB in the skin of different leprosy patients. Scale bars, 100 µm. **C** Secretion level of GPNMB ECD in the sera of different leprosy patients, *n* (L-Lep) = 10, *n* (T-Lep) = 5. **D** qPCR analysis of *IL-6*, *TNF-α*, and *IL-1β* mRNA levels in sgCtrl or *Gpnmb*-knockout THP-1 cells infected with *M. leprae* for the indicated times; *n* = 3. **E** ELISA quantification of IL-6, TNF-α, and IL-1β secretion levels in sgCtrl- or *Gpnmb*-knockout THP-1 cells infected with *M. leprae* for the indicated times; *n* = 3. **F** qPCR analysis of *IL-6*, *TNF-α*, and *IL-1β* mRNA levels in sgCtrl- or *Gpnmb*-knockout THP-1 cells infected with *M. marinum* for the indicated times; *n* = 3. The data are shown as the means ± SDs and were analyzed via an unpaired two-tailed *t* test (*p < 0.05, **p < 0.01, ***p < 0.001)

As a key part of innate immunity and a major host of leprosy pathogens, macrophage dysfunction is an important reason for *M. leprae* accumulation in L-Lep. Nevertheless, the underlying mechanism remains unclear. To identify the key regulator of *M. leprae* escape from host immune defenses in L-Lep, we previously performed single-cell RNA sequencing [28] and reported that the mRNA level of *GPNMB* was significantly greater in L-Lep than in T-Lep. The expression level of *GPNMB* in publicly available gene array data from the NCBI GEO database (accession number: GSE17763) confirmed that GPNMB was upregulated in the skin of L-Lep patients (Fig. 1A). To further confirm these findings, multiple immunohistochemistry (mIHC) was used. mIHC images revealed that the protein level of GPNMB in L-Lep patient skin was much greater than that in T-Lep skin (Fig. 1B). Previous reports have shown that the extracellular portion (ECD) of GPNMB can be shed from the cell surface, thus releasing soluble fragments [29]. We monitored the concentration of the GPNMB ECD in the serum of patients with leprosy and detected a higher ECD concentration in the serum of patients with L-Lep than in those with T-Lep (Fig. 1C).

Compared with patients with T-Lep, patients with L-Lep presented weaker antimicrobial innate immune responses, accompanied by lower proinflammatory cytokine levels and massive proliferation of *M. leprae* in macrophages [9]. To investigate the role of GPNMB in antibacterial immunity, we infected THP-1 cells with *M. leprae*. We found that the expression levels and secretion levels of IL-6, TNF-α and IL-1β were greater in *Gpnmb*-deficient THP-1 cells than in sgCtrl THP-1 cells upon bacterial infection (Fig. 1D, E). Consistent with the findings in *M. leprae*, *M. marinum*-infected *Gpnmb*-knockout and -knockdown (Supplementary Fig. S1A) THP-1 cells presented elevated levels of IL-6, TNF-α and IL-1β (Fig. 1F and Supplementary Fig. S1B). These data indicated that GPNMB suppressed the production of proinflammatory cytokines upon bacterial infection.

### GPNMB is important for maintaining bacterial proliferation in vivo

**Fig. 2:**
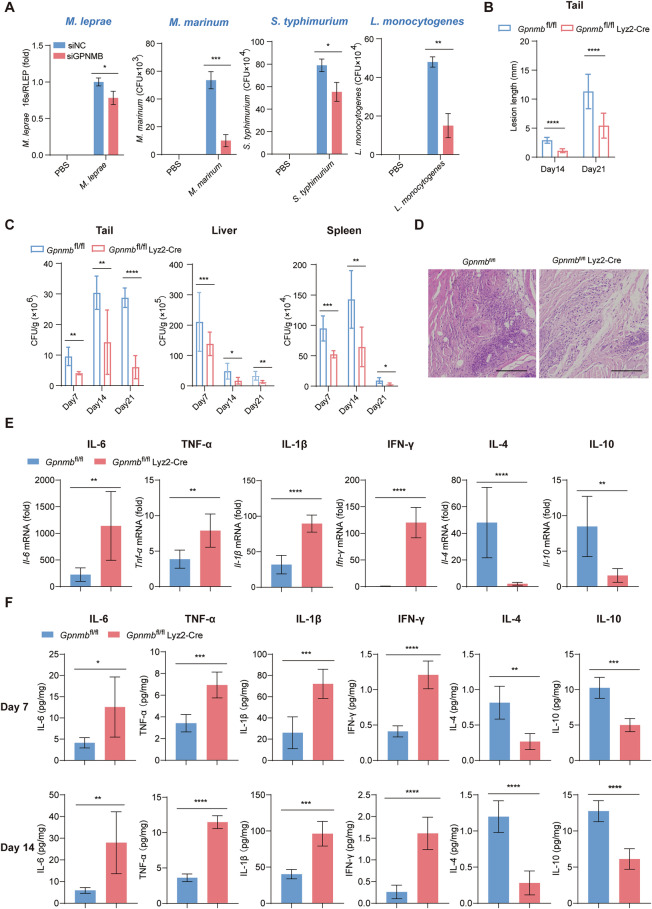
Loss of GPNMB restricts the proliferation of bacteria in vivo. **A** MDMs were transfected with negative control (siNC) or *GPNMB* siRNA (siGPNMB) for 24 h, followed by infection with *M. leprae* (MOI of 10)*, M. marinum* (MOI of 10)*, S. typhimurium* (MOI of 1) *or L. monocytogenes* (MOI of 1) for different durations. The viability of *M. leprae* in MDMs was calculated by the ratio of bacterial 16S rRNA and DNA (RLEP) detected by qPCR after infection for 24 h. The number of intracellular *M. marinum* at 24 h post infection*, S. typhimurium* at 5 h post infection or *L. monocytogenes* at 5 h post infection was determined by colony counting, *n* = 3. **B** Lengths of visible lesions on the tails of *Gpnmb*^fl/fl^ or *Gpnmb*^fl/fl^ Lyz2-Cre mice after intravenous injection of *M. marinum* (2 × 10^7^ CFU per mouse) for 14 and 21 days, *n* = 9. **C** *Gpnmb*^fl/fl^ or *Gpnmb*^fl/fl^ Lyz2-Cre mice were sacrificed 7, 14 or 21 days post infection with *M. marinum* (2 × 10^7^ CFU per mouse). Tails, livers, and spleens were homogenized and plated on Middlebrook 7H9 plates (*n* = 5). **D** Hematoxylin‒eosin staining of tail sections from *Gpnmb*^fl/fl^ or *Gpnmb*^fl/fl^ Lyz2-Cre mice 14 days post infection with *M. marinum* (2 × 10^7^ CFU per mouse). Scale bars, 100 µm. **E** qPCR analysis of *Il-6*, *Tnf-α*, *Il-1β, Ifn-γ, Il-4* and *Il-10* mRNA levels in the tails of *Gpnmb*^fl/fl^ or *Gpnmb*^fl/fl^ Lyz2-Cre mice 7 days post infection with *M. marinum* (2 × 10^7^ CFU per mouse), *n* = 7. **F** ELISA analysis of IL-6, TNF-α, IL-1β, IFN-γ, IL-4 and IL-10 secretion levels in the tails of *Gpnmb*^fl/fl^ or *Gpnmb*^fl/fl^ Lyz2-Cre mice 7 or 14 days post infection with *M. marinum* (2 × 10^7^ CFU per mouse), *n* = 5. The data are shown as the means ± SDs and were analyzed via an unpaired two-tailed *t* test (*p < 0.05, **p < 0.01, ***p < 0.001, ****p < 0.0001)

To examine the effect of GPNMB on bacterial survival in macrophages, we prepared human peripheral blood monocyte-derived macrophages (MDMs) and knocked down the expression of GPNMB (Supplementary Fig. S1C). *Gpnmb*-deficient cells showed diminished viability of *M. leprae*, *M. marinum*, *Salmonella Typhimurium* (*S. typhimurium*), and *Listeria monocytogenes* (*L. monocytogenes*) after infection (Fig. 2A). Similarly, compared with sgCtrl THP-1 cells, *Gpnmb*-knockout THP-1 cells presented a lower bacterial burden after *M. leprae* or *M. marinum* infection for 24 h (Supplementary Fig. S2A, B). To further confirm the function of GPNMB in vivo, we intravenously injected *Gpnmb*^fl/fl^ and *Gpnmb*^fl/fl^ Lyz2-Cre mice with *M. marinum*. Compared with *Gpnmb*^fl/fl^ Lyz2-Cre mice, *Gpnmb*^fl/fl^ mice presented severe tail disease at days 14 and 21 post infection with *M. marinum* (Fig. 2B and Supplementary Fig. S2C). Furthermore, the *M. marinum* burden in the tails, livers and spleens was greater in Gpnmbfl/fl mice than in *Gpnmb*^fl/fl^ Lyz2-Cre mice (Fig. 2C). Severe injuries and increased infiltration of monocytes were observed in the tails of *Gpnmb*^fl/fl^ mice after *M. marinum* infection for 14 days compared with those in the tails of *Gpnmb*^fl/fl^ Lyz2-Cre mice (Fig. 2D). Moreover, consistent with the results in the leprosy patients and cell infection model, *Gpnmb*^fl/fl^ Lyz2-Cre mice infected with *M. marinum* expressed and secreted more pro-inflammatory cytokines, such as IL-6, TNF-α, IL-1β and IFN-γ, and fewer anti-inflammatory cytokines, such as IL-4 and IL-10, both on day 14 and day 21 post infection (Fig. 2E, F). Overall, we demonstrated that GPNMB inhibited antibacterial immune responses in vivo.

### GPNMB disrupts autophagic flux in cells

GPNMB is a transmembrane glycoprotein; therefore, we wondered whether GPNMB suppresses the host immune response by mediating macrophage phagocytosis. However, we observed no difference between sgCtrl and *Gpnmb*-knockout THP-1 cells in phagocytosis assays with the latex bead-rabbit IgG-FITC complex (Supplementary Fig. S3).

**Fig. 3:**
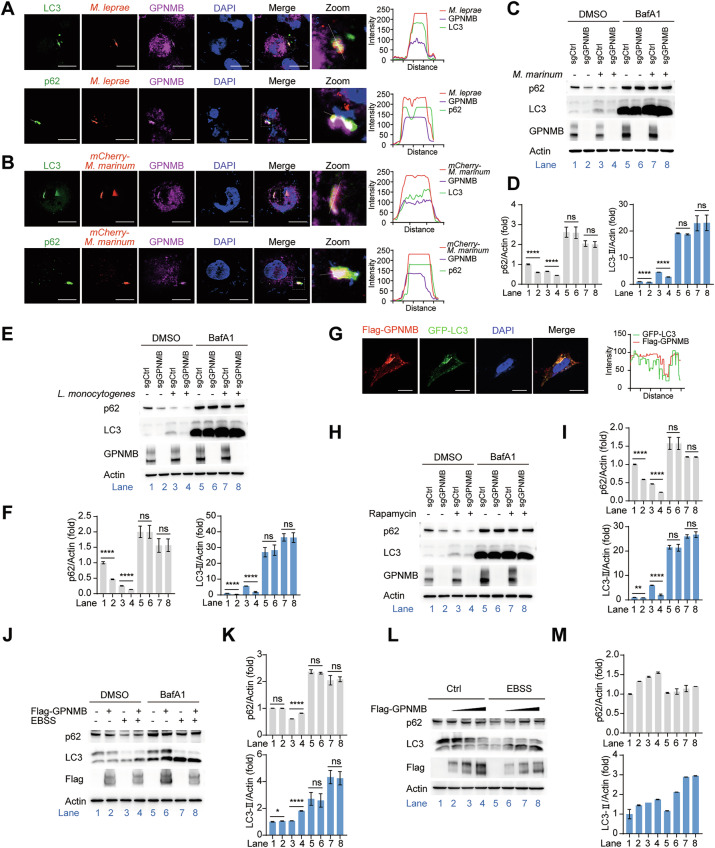
GPNMB inhibits cellular autophagic flux. **A** Confocal microscopy of THP-1 cells infected with red fluorescence-labeled *M. leprae* (MOI of 10) for 6 h. The cells were stained with antibodies against GPNMB (purple), LC3 (green), p62 (green) and the DNA-binding dye DAPI (blue). Scale bar, 10 μm. **B** Confocal microscopy of THP-1 cells infected with mCherry*-M. marinum* (MOI of 10) for 6 h. The cells were stained with antibodies against GPNMB (purple), LC3 (green), p62 (green) and the DNA-binding dye DAPI (blue). Scale bar, 10 μm. **C** Western blot analysis of p62 and LC3 protein levels in sgCtrl or *Gpnmb*-knockout THP-1 cells infected with *M. marinum* (MOI of 10) for 0–6 h, followed by bafilomycin A1 (BafA1, 1 μM) treatment for 6 h. **D** Quantification of the relative p62 (left) and LC3-II (right) protein levels in (**C**). **E** Western blot analysis of p62 and LC3-II protein levels in sgCtrl- or *Gpnmb*-knockout THP-1 cells infected with *L. monocytogenes* (1 MOI) for 0–6 h, followed by bafilomycin A1 (BafA1, 1 μM) treatment for 6 h. **F** Quantification of the relative p62 (left) and LC3-II (right) protein levels in (**E**). **G** Confocal microscopy of HEK293T cells transfected with Flag-GPNMB and GFP-LC3 for 24 h. The cells were stained with antibodies specific for Flag (red) and the DNA-binding dye DAPI (blue). Scale bar, 10 μm. **H** Western blot analysis of p62 and LC3-II protein levels in sgCtrl- or *Gpnmb*-knockout THP-1 cells stimulated with rapamycin (200 ng/ml) for 0–6 h, followed by bafilomycin A1 (BafA1, 1 μM) treatment for 6 h. **I** Quantification of relative p62 (top) and LC3-II (bottom) protein levels in (**H**). **J** Western blot analysis of p62 and LC3-II protein levels in HEK293T cells transfected with Ctrl or Flag-GPNMB expression plasmids for 24 h, followed by EBSS treatment for 0–6 h and then treatment with bafilomycin A1 (BafA1, 1 μM) for 6 h. **K** Quantification of relative p62 (top) and LC3-II (bottom) protein levels in (**J**). **L** Western blot analysis of p62 and LC3 protein levels in HEK293T cells transfected with the Flag-GPNMB expression plasmid for 24 h, followed by EBSS treatment for 6 h. **M** Quantification of relative p62 (top) and LC3-II (bottom) protein levels upon EBSS treatment in (**L**). The data are shown as the means ± SDs and were analyzed via an unpaired two-tailed *t* test (*p < 0.05, **p < 0.01, ****p < 0.0001; ns not significant)

GPNMB expression is regulated by melanogenesis-associated transcription factor (MITF), a member of the MiT/TFE subfamily [30]. This transcription factor family is well known to regulate the expression of proteins involved in autophagosome and lysosome biogenesis [31]. To investigate whether GPNMB suppressed antibacterial immunity by disrupting autophagy, we monitored the localization of GPNMB upon *M. leprae* infection. LC3-II and p62 are two important indicators of autophagy progression [32, 33]. After infection for 6 h, GPNMB colocalized with *M. leprae* in the LC3^+^ or p62^+^ cytoplasmic puncta (Fig. 3A and Supplementary Fig. S4A). Consistently, GPNMB was also detected in the LC3^+^ or p62^+^ cytoplasmic puncta upon *M. marinum* infection (Fig. 3B), which suggested that GPNMB was involved in bacteria-mediated autophagy (xenophagy).

Upon *M. marinum* infection, the levels of both p62 and LC3-II were decreased in the *Gpnmb*-knockout THP-1 cells, indicating that autophagic flux was impaired (Fig. 3C, D). Similar phenomena also occurred upon *L. monocytogenes* infection (Fig. 3E, F). These findings indicated that GPNMB disrupted xenophagy upon bacterial infection.

Next, we examined whether GPNMB has similar effects on other forms of autophagy, such as nonselective autophagy. Confocal microscopy images revealed that GPNMB colocalized with LC3 in HEK293T cells (Fig. 3G and Supplementary Fig. S4B). The western blot results revealed decreased protein levels of p62 and LC3-II in *Gpnmb*-knockout THP-1 cells after stimulation with rapamycin (Fig. 3H, I). Then, we mimicked starvation conditions through Earle’s balanced salt solution (EBSS) treatment. We found that starvation robustly increased the degradation of p62 (Fig. 3J–M), whereas p62 degradation was abolished in HEK293T cells overexpressing GPNMB (Fig. 3J–M). Moreover, we found that GPNMB promoted the accumulation of LC3-II upon EBSS treatment (Fig. 3J–M). Taken together, our data demonstrated that GPNMB blocked autophagic flux via both selective autophagy and nonselective autophagy.

### Fusion of autophagosomes with lysosomes is blocked by GPNMB

**Fig. 4:**
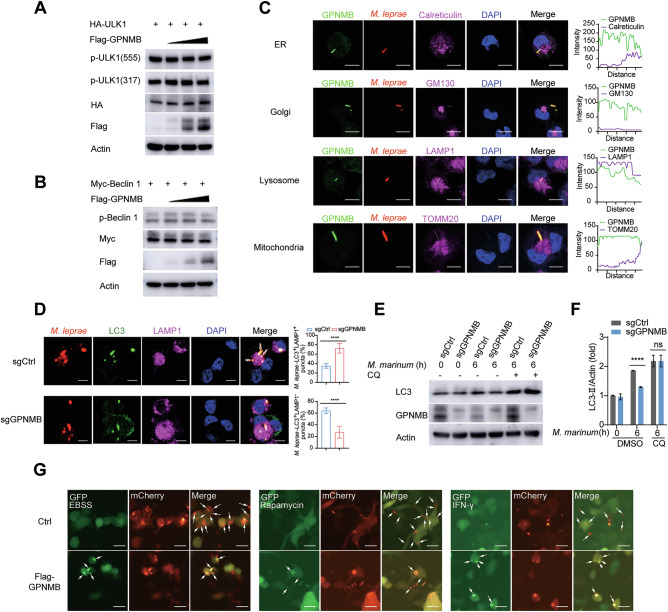
GPNMB blocks the maturation of autophagosomes. **A** Western blot analysis of the phosphorylation level of ULK1 in HEK293T cells transfected with HA-ULK1 and Flag-GPNMB expression plasmids for 24 h. **B** Western blot analysis of the phosphorylation level of Beclin 1 in HEK293T cells transfected with Myc-Beclin 1 and Flag-GPNMB expression plasmids for 24 h. **C** Confocal microscopy of THP-1 cells infected with red fluorescence-labeled *M. leprae* (MOI of 10) for 6 h. Cells were stained with antibodies specific for GPNMB (green), calreticulin (ER marker, purple), GM130 (Golgi marker, purple), LAMP1 (lysosome marker, purple), TOMM20 (mitochondria marker, purple) and the DNA-binding dye DAPI (blue). Scale bar, 5 μm. **D** Confocal microscopy of sgCtrl- or *Gpnmb*-knockout THP-1 cells infected with red fluorescence-labeled *M. leprae* (MOI of 10) for 6 h. The cells were stained with antibodies against LC3 (green), LAMP1 (purple), and the DNA-binding dye DAPI (blue). Scale bar, 5 μm. **E** Western blot analysis of the LC3 protein level in sgCtrl or *Gpnmb*-knockout THP-1 cells pretreated with chloroquine (CQ, 50 μM) for 2 h and then transfected with *M. marinum* (MOI of 10) for 0–6 h. **F** Quantification of the relative LC3-II protein level in (**E**). **G** Confocal microscopy of HEK293T cells transfected with GFP-mCherry-LC3 and Flag-GPNMB expression plasmids for 24 h, followed by stimulation with EBSS, rapamycin (200 ng/ml) or IFN-γ (100 ng/ml) for 24 h. Scale bar, 20 μm. The data are shown as the means ± SDs and were analyzed via an unpaired two-tailed *t* test (****p < 0.0001; ns not significant)

We next determined at which step the autophagy pathway is impaired by GPNMB. At the beginning of autophagy, ULK1 is phosphorylated [34, 35] and forms a protein complex with several partners, such as the ULK1/FIP200/ATG13 Atg1/ULK1 complex. The ULK1 complex subsequently activates the VPS34/Beclin 1/ATG14L PI (3)P kinase complex [36] to promote phagophore formation and expansion [32, 37]. We evaluated the phosphorylation of ULK1 and Beclin 1 after the overexpression of GPNMB in HEK293T cells. We found that the phosphorylation levels of ULK1 and Beclin 1 were not affected by GPNMB overexpression, indicating that GPNMB has no effect on autophagosome formation (Fig. 4A, B). Through confocal microscopy, we identified the colocalization of GPNMB with the lysosomal marker lysosomal-associated membrane protein 1 (LAMP1) upon *M. leprae* infection (Fig. 4C and Supplementary Fig. S4C) but not with the endoplasmic reticulum marker calreticulin, the Golgi body marker Golgi matrix protein 130 (GM130), or the mitochondrial marker translocase of the outer mitochondrial membrane 20 (TOMM20) (Fig. 4C), suggesting that GPNMB may be involved in the maturation of autophagosomes. GPNMB inhibited the fusion of *M. leprae*-LC3^+^ vacuoles with LAMP1-labeled lysosomes in sgCtrl THP-1 cells (Fig. 4D). Moreover, the amount of LC3-II in the *Gpnmb*-knockout THP-1 cells recovered upon treatment with chloroquine (CQ, a drug used to inhibit lysosomal degradation) (Fig. 4E, F) or bafilomycin A1 (an autophagy inhibitor at the late stage) (Fig. 3C–F, H, I). Moreover, we measured the characteristics of lysosomes upon GPNMB overexpression or deficiency. We found that the acidic environment and the intracellular activity of lysosomes were not affected by GPNMB (Supplementary Fig. S5A–C). Taken together, these findings indicated that GPNMB blocked the fusion of autophagosomes with lysosomes (autophagosome maturation) without affecting the production of LC3-II.

To confirm this finding, we transfected a GFP-mCherry-LC3 expression plasmid into HEK293T cells and treated the cells with EBSS, rapamycin, or IFN-γ to induce autophagy. Since the green fluorescence of the fusion protein is very sensitive to the acidic environment of lysosomes and quickly quenches in autolysosomes, only red fluorescence could be detected in the autolysosomes [32]. As expected, we found that the maturation of autophagosomes was disrupted in GPNMB-overexpressing HEK293T cells after EBSS, rapamycin or IFN-γ treatment (Fig. 4G).

### GPNMB disrupts autophagosome maturation through binding to STX17

**Fig. 5:**
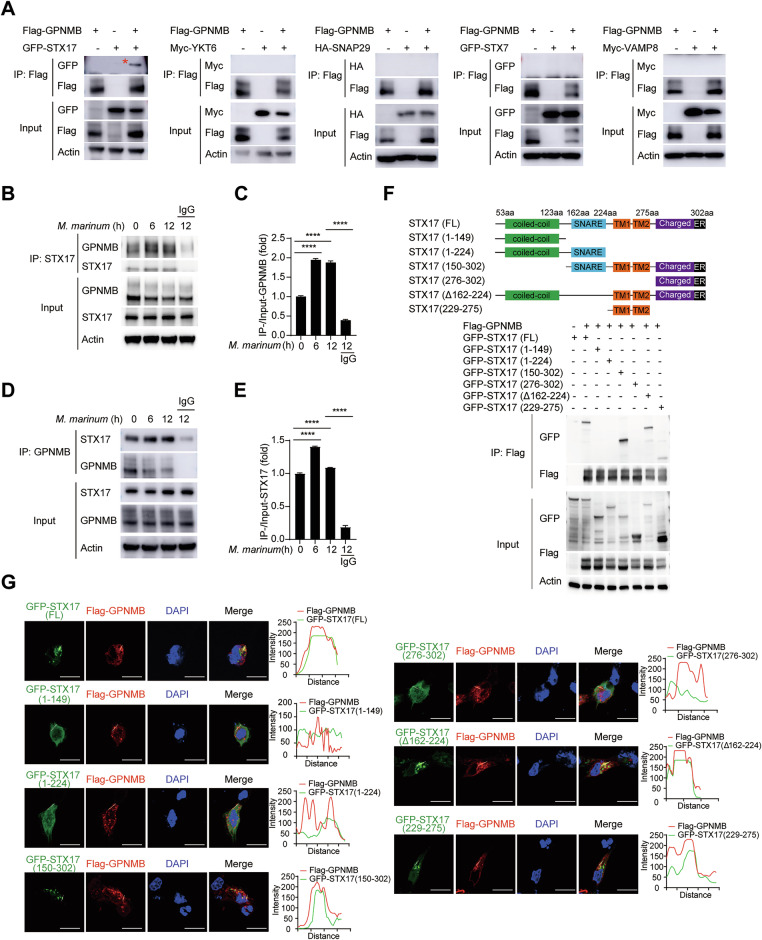
GPNMB targets STX17. **A** HEK293T cells were transfected with plasmids expressing GFP-STX17, Myc-YKT6, HA-SNAP29, GFP-STX7, or Myc-VAMP8 together with Flag-GPNMB for 24 h. Co-IP and immunoblotting were performed with the indicated antibodies. **B** Co-IP and immunoblotting of GPNMB with STX17 in THP-1 cells infected with *M. marinum* (MOI of 10) for 0–12 h. As a negative control for Co-IP, the cell lysates were treated with normal IgG instead of the indicated antibody. **C** Quantification of the relative GPNMB protein level upon immunoprecipitation with the STX17 antibody in (**B**). **D** Co-IP and immunoblotting of GPNMB with STX17 in THP-1 cells infected with *M. marinum* (MOI of 10) for 0–12 h. As a negative control for Co-IP, the cell lysates were treated with normal IgG instead of the indicated antibody. **E** Quantification of the relative STX17 protein level upon immunoprecipitation with the GPNMB antibody in (**D**). **F** HEK293T cells were transfected with plasmids expressing GFP-STX17 or its truncations together with Flag-GPNMB for 24 h. Co-IP and immunoblotting were performed with the indicated antibodies. **G** Confocal microscopy of HEK293T cells transfected with plasmids expressing GFP-STX17 or its truncations together with Flag-GPNMB for 24 h. The cells were stained with antibodies against Flag (red) and the DNA-binding dye DAPI (blue). Scale bar, 10 μm. The data are shown as the means ± SDs and were analyzed via an unpaired two-tailed *t* test (***p < 0.001, ****p < 0.0001)

The fusion of autophagosomes with lysosomes is mediated by the assembly of the STX17-SNAP29-VAMP8 or YKT6-SNAP29-STX7 SNARE complex [5]. To investigate the target protein of GPNMB, we cotransfected Flag-GPNMB with GFP-STX17, Myc-YKT6, HA-SNAP29, GFP-STX7, or Myc-VAMP8 expression plasmids into HEK293T cells. Coimmunoprecipitation (co-IP) experiments revealed that GPNMB specifically bound to STX17 rather than other components of the SNARE complex (Fig. 5A). Moreover, the binding of GPNMB with STX17 was enhanced upon bacterial infection (Fig. 5B–E and Supplementary Fig. S6A). To ensure the specificity of protein‒protein binding, we used nonspecific IgG as a negative control.

STX17 has two tandem transmembrane (TM) domains near the C-terminal end, which are essential for its association with the autophagosomal membrane [6, 38]. To investigate which domain of STX17 is essential for its binding with GPNMB, we constructed six STX17 truncation mutants (Fig. 5F) and transfected them together with a Flag-GPNMB expression plasmid into HEK293T cells. We found that depletion of the STX17 TM domain disrupted the interaction between STX17 and GPNMB (Fig. 5F, G), which indicated that the autophagosomal localization of STX17 was essential for the ability of GPNMB to bind with. A previous study revealed that STX17 is present only on completely closed autophagosomes [6]. Thus, we speculated that GPNMB is likely to move to autophagosomes during infection.

### GPNMB blocks the interaction between STX17 and SNAP29

**Fig. 6:**
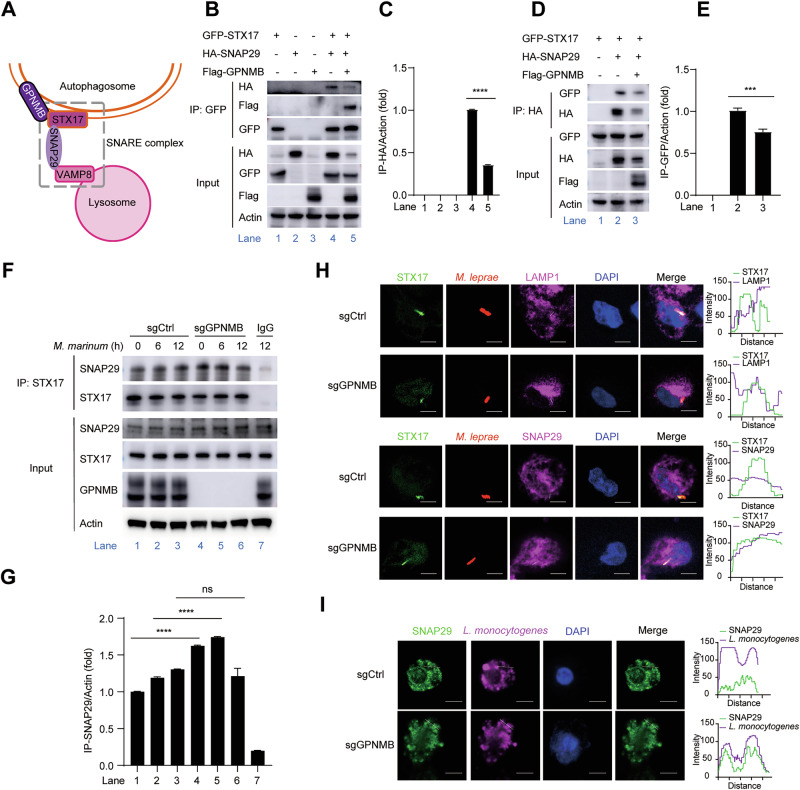
GPNMB disrupts the interaction of STX17 with SNAP29. **A** The SNARE complex mediates the fusion of autophagosomes with lysosomes. **B** HEK293T cells were transfected with GFP-STX17 or HA-SNAP29 together with the Flag-GPNMB expression plasmid for 24 h. Co-IP and immunoblotting were performed with the indicated antibodies. **C** Quantification of the relative HA-SNAP29 protein level upon immunoprecipitation with the GFP antibody in (**B**). **D** HEK293T cells were transfected with GFP-STX17 or HA-SNAP29 together with the Flag-GPNMB expression plasmid for 24 h. Co-IP and immunoblotting were performed with the indicated antibodies. **E** Quantification of the relative GFP-STX17 protein level upon immunoprecipitation with the HA antibody in (**D**). **F** Co-IP and immunoblotting of GPNMB with SNAP29 in sgCtrl- or *Gpnmb*-knockout THP-1 cells infected with *M. marinum* (MOI of 10) for 0–12 h. As a negative control for Co-IP, the cell lysates were treated with normal IgG instead of the indicated antibody. **G** Quantification of the relative SNAP29 protein level upon immunoprecipitation with the STX17 antibody in (**F**). **H** Confocal microscopy of THP-1 cells infected with red fluorescence-labeled *M. leprae* (MOI of 10) for 6 h. The cells were stained with antibodies against STX17 (green), LAMP1 (purple), SNAP29 (purple) and the DNA-binding dye DAPI (blue). Scale bar, 5 μm. **I** Confocal microscopy of THP-1 cells infected with *L. monocytogenes* (1 MOI) for 6 h. The cells were stained with antibodies against SNAP29 (green) and *L. monocytogenes* (purple) and the DNA-binding dye DAPI (blue). Scale bar, 5 μm. The data are shown as the means ± SDs and were analyzed via an unpaired two-tailed *t* test (***p < 0.001, ****p < 0.0001; ns no significance)

Next, we determined whether GPNMB disrupted the assembly of the STX17-SNAP29-VAMP8 complex (Fig. 6A) to inhibit the fusion of autophagosomes with lysosomes. Co-IP results revealed that GPNMB inhibited the interaction of STX17 and SNAP29 in HEK293T cells transfected with the GFP-STX17 and HA-SNAP29 expression plasmids in the presence of Flag-GPNMB (Fig. 6B–E). Consistently, in *Gpnmb*-deficient THP-1 cells, STX17 bound more tightly to SNAP29 upon *M. leprae* or *M. marinum* infection (Fig. 6F–H). Furthermore, we found that the fusion of *M. leprae* containing STX17^+^ autophagosomes with LAMP1^+^ lysosomes was greater in *Gpnmb*-knockout THP-1 cells than in sgCtrl THP-1 cells (Fig. 6H and Supplementary Fig. S6B). Similarly, the colocalization of SNAP29 and *L. monocytogenes* was lower in sgCtrl THP-1 cells than in *Gpnmb*-knockout THP-1 cells (Fig. 6I). Taken together, our data revealed that GPNMB blocked cellular autophagic flux by disrupting the interaction between STX17 and SNAP29 during bacterial infection.

### Deglycosylation of GPNMB promotes the degradation of SNAP29

**Fig. 7:**
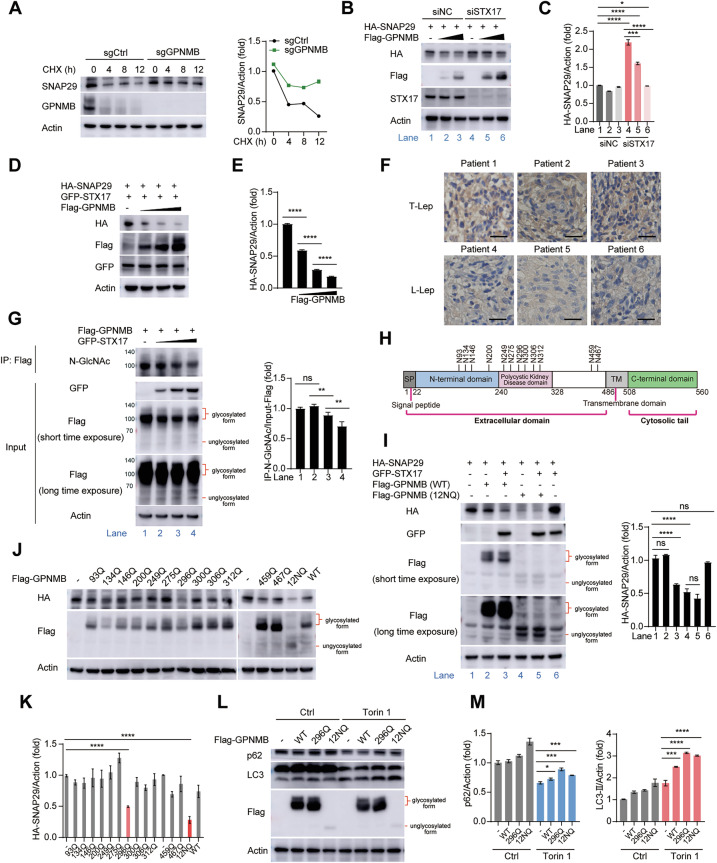
Deglycosylated GPNMB promotes the degradation of SNAP29. **A** Western blot analysis of SNAP29 protein levels in sgCtrl or *Gpnmb*-knockout THP-1 cells infected with *M. marinum* (MOI of 10) for 6 h, followed by cycloheximide (CHX, 100 μg/ml) treatment for 0–12 h (left). Densitometric quantification was performed via ImageJ (right). **B** Western blot analysis of HA-SNAP29 protein levels in HEK293T cells transfected with control (siNC) or STX17 siRNA (siSTX17) for 24 h, followed by HA-SNAP29 and Flag-GPNMB expression plasmid transfection for 24 h. **C** Quantification of the relative HA-SNAP29 protein level in (**B**). **D** Western blot analysis of HA-SNAP29 protein levels in HEK293T cells transfected with HA-SNAP29, GFP-STX17 and Flag-GPNMB expression plasmids for 24 h. **E** Quantification of the relative HA-SNAP29 protein level in (**D**). **F** Immunohistochemistry of SNAP29 in the skin of different leprosy patients, *n* = 3. Scale bar, 25 μm. **G** Co-IP and western blot analysis of the glycosylation level of GPNMB in HEK293T cells transfected with Flag-GPNMB and GFP-STX17 expression plasmids for 24 h (left). Densitometric quantification was performed via ImageJ (right). **H** Structure and glycosylation sites of GPNMB. **I** Western blot analysis of HA-SNAP29 protein levels in HEK293T cells transfected with HA-SNAP29 or GFP-STX17 together with Flag-GPNMB (WT) or Flag-GPNMB (12NQ) expression plasmids for 24 h (left). Densitometric quantification was performed via ImageJ (right). **J** Western blot analysis of HA-SNAP29 protein levels in HEK293T cells transfected with HA-SNAP29 together with Flag-GPNMB (WT) or Flag-GPNMB mutation expression plasmids for 24 h. **K** Quantification of the relative HA-SNAP29 protein level in (**J**). **L** Western blot analysis of p62 and LC3 protein levels in HEK293T cells transfected with Flag-GPNMB (WT) or Flag-GPNMB mutation expression plasmids for 24 h, followed by Torin 1 (500 μM) treatment for 4 h. **M** Quantification of relative p62 (left) and LC3-II (right) protein levels in (**L**). The data are shown as the means ± SDs and were analyzed via an unpaired two-tailed *t* test (*p < 0.05, *p < 0.01, ***p < 0.001, ****p < 0.0001; ns not significant)

In addition to the finding that GPNMB disrupted the interaction of STX17 with SNAP29, the overexpression of GPNMB seemed to promote the degradation of SNAP29 (Fig. 6B, D). To confirm this finding, we treated THP-1 cells with the de novo protein synthesis inhibitor cycloheximide (CHX). We found that the degradation rate of SNAP29 was decreased in *Gpnmb*-knockout cells (Fig. 7A). Given that GPNMB cannot bind with SNAP29 directly (Fig. 5A), we wondered whether GPNMB promoted the degradation of SNAP29 through STX17. As expected, we found that GPNMB bound to the STX17-SNAP29 complex (Supplementary Fig. S7A) and promoted the degradation of SNAP29 in a STX17-dependent manner (Fig. 7B, C). The protein level of SNAP29 was greater in *Stx17*-deficient HEK293T cells than in control cells (Fig. 7B, C and Supplementary Fig. S7B). GPNMB promoted the degradation of SNAP29 in a dose-dependent manner in the presence of STX17 (Fig. 7D, E). More interestingly, we found that the protein level of SNAP29 was lower in patients with L-Lep than in those with T-Lep (Fig. 7F), which was consistent with our cell experiment results.

Next, we investigated how GPNMB promoted STX17-mediated SNAP29 degradation. GPNMB is a highly glycosylated transmembrane protein. A previous study showed that depleting its glycosylation alters cellular downstream signaling [39]. Furthermore, upon infection, bacterial pathogens can modulate the glycosylation of host proteins to facilitate pathogenesis by inducing abnormal host protein activity and abundance [40]. Thus, we wondered whether the glycosylation level of GPNMB was changed upon binding with STX17. As expected, through co-IP and immunoblot analysis, we found that STX17 decreased the glycosylation level of GPNMB in a dose-dependent manner (Fig. 7G). GPNMB has 12 glycosylation sites in its ECD domain [25] (Fig. 7H). We mutated all 12 amino acids to glutamine to eliminate the glycosylation of GPNMB to study the importance of GPNMB glycosylation to SNAP29 protein stability. N-linked glycosylation is a posttranslational modification that is crucial for membrane protein folding, stability and signal transduction [41, 42]. Compared with their unglycosylated counterparts, N-glycans can stabilize the conformation of the secondary structure [43] of glycoproteins and enhance overall protein stability and resistance to proteolysis [44, 45]. In this study, we found that the expression level of GPNMB (12NQ) was much lower than that of WT GPNMB (Fig. 7I). However, GPNMB (12NQ) overexpression significantly reduced the protein level of SNAP29, which indicated that the glycosylation status of GPNMB was closely related to the protein level of SNAP29 (Fig. 7I). More importantly, our results revealed that GPNMB (12NQ) decreased the protein level of SNAP29 independently of STX17 (Fig. 7I).

To identify asparagine glycosylated residues in GPNMB, which are essential for the degradation of SNAP29, we mutated each of the 12 asparagine residues to glutamine and cotransfected the Flag-GPNMB mutant and HA-SNAP29 expression plasmids together into HEK293T cells. Western blot analysis revealed that the protein level of SNAP29 was significantly decreased in the Flag-GPNMB^296Q-^ or Flag-GPNMB^12NQ^-transfected HEK293T cells (Fig. 7J, K), which indicated that the glycosylation status of GPNMB at N296 was closely related to the protein level of SNAP29. Next, we examined the effect of GPNMB N296 glycosylation on autophagy. We overexpressed GPNMB, GPNMB^296Q^, or GPNMB^12NQ^ in HEK293T cells. Twenty-four hours later, Torin 1, which stimulates autophagy, was added to the cell culture medium. The results of the western blot analysis revealed that the autophagic flux of the GPNMB^296Q-^ and GPNMB^12NQ^-overexpressing HEK293T cells was more severely damaged than that of the WT GPNMB cells (Fig. 7L, M). Therefore, we concluded that the deglycosylation of N296 by GPNMB may inhibit xenophagy and promote bacterial survival during infection.

## Discussion

Impaired autophagic flux is closely related to the accumulation of invading microbes in macrophages. In this study, we revealed that GPNMB inhibited host defense against intracellular bacterial infection by disrupting the fusion of autophagosomes with lysosomes. Upon bacterial infection, GPNMB is recruited to autophagosomes through interaction with STX17, disrupting the assembly of the STX17-SNAP29-VAMP8 SNARE complex. Furthermore, binding to STX17 promoted the deglycosylation of GPNMB at N296, leading to the degradation of SNAP29. In human macrophages and a mouse infection model, *the* loss of *Gpnmb* increased host resistance to bacterial infection. This research therefore revealed a novel mechanism through which GPNMB impairs the autophagic process and consequently facilitates bacterial survival within macrophages.

GPNMB is a glycosylated type I transmembrane protein [25, 46]. Previous studies have demonstrated the important roles of GPNMB in host innate and adaptive immunity. GPNMB can promote the polarization of macrophages toward the M2 phenotype [46, 47], suppress proinflammatory cytokine production upon LPS stimulation [27], and enhance tumor migration and invasion in cancer [46, 48, 49]. However, the function of GPNMB in microbial infection has rarely been studied. During *Trichophyton rubrum* or *Microsporum audouinii* infection, GPNMB has been found to bind to the cell walls of these fungi and subsequently induce dendritic cell activation [17]. In the case of porcine reproductive and respiratory syndrome virus (PRRSV) infection, GPNMB inhibits viral replication by preventing autophagosome‒lysosome fusion [50]. However, the molecular mechanism has not been elucidated. In this study, GPNMB overexpression led to the accumulation of bacteria in macrophages. Similar to the findings in viral infection, we also noted that GPNMB disrupted the fusion of autophagosomes with lysosomes upon bacterial infection. Moreover. We found that GPNMB blocked the assembly of the STX17-SNAP29-VAMP8 SNARE complex, thereby inhibiting autophagosome maturation during bacterial infection.

Increasing evidence suggests a complex interplay between inflammation and autophagy. Some studies have shown that autophagy can downregulate proinflammatory cytokine production in macrophages [51, 52], whereas others have demonstrated that autophagy can either up- or downregulate proinflammatory responses depending on the cellular context [53, 54]. In this study, we found that GPNMB suppressed the production of proinflammatory cytokines upon bacterial infection. Although GPNMB may disrupt cellular xenophagy to inhibit proinflammatory cytokine production, we believe that GPNMB regulates host antibacterial immunity through multiple pathways. Notably, we found that GPNMB inhibited the activation of the NF-κB signaling pathway during *L. monocytogenes* infection (Supplementary Fig. S8).

The involvement of the STX17-SNAP29-VAMP8 and YKT6-SNAP29-STX7 SNARE complexes in the fusion of autophagosomes with lysosomes has been well established, but the differences between these two sets of SNARE complexes in modulating this fusion remain unclear. Whether these complexes work synergistically to increase autophagosome‒lysosome fusion efficiency or function independently in different forms of autophagy has not been definitively determined. One study revealed the independent actions of these complexes [7], whereas another study demonstrated that YKT6 initially forms a priming complex with STX17 and SNAP29, which is then replaced by VAMP8 to form STX17-SNAP29-VAMP8 [8]. However, in both studies, the presence of the STX17-SNAP29-YKT6 complex was observed in autophagosomes, indicating potential crosstalk between these two SNARE complexes. In this study, we demonstrated that GPNMB is recruited to autophagosomes through interactions with autophagosomally localized STX17 rather than YKT6. Although YKT6 and STX17 reportedly form a stable complex in autophagosomes [8], YKT6 is not essential for the autophagosomal localization of GPNMB. Collectively, our findings and those of other researchers suggested that the STX17-SNAP29-VAMP8 complex seems to play a predominant role in xenophagy [55, 56]. Similarly, various invading pathogens, such as *Legionella pneumophila* [57], *Streptococcus pneumoniae* [58] and SARS-CoV-2 [59], have evolved strategies to evade xenophagy by manipulating the assembly of the STX17-SNAP29-VAMP8 complex rather than the YKT6-SNAP29-STX7 SNARE complex, allowing them to survive within intracellular replicative niches.

After interacting with STX17, GPNMB binds to SNAP29 on autophagosomes and promotes the degradation of SNAP29. Since SNAP29 is a common component of both types of SNARE complexes, GPNMB, in addition to its role in xenophagy, is likely to play important roles in other forms of autophagy. Here, we demonstrated that GPNMB suppressed cellular autophagic flux in both selective and nonselective autophagy by blocking the fusion of autophagosomes with lysosomes. Furthermore, the glycosylation status of GPNMB determines the fate of xenophagy. Deglycosylation of GPNMB at residue N296 led to the degradation of SNAP29 upon bacterial infection. Therefore, determining how to maintain the glycosylation of N296 by GPNMB may be a new direction for antibacterial drug development.

In addition to combating infections, macrophages play critical roles in tumorigenesis. Tumor-associated macrophages (TAMs) are pivotal in mediating antitumor activity through processes such as phagocytosis [60]. Targeting signaling pathways to reprogram TAMs, such as enhancing tumor cell phagocytosis [61], has emerged as a promising strategy for cancer treatment. Notably, high GPNMB expression in TAMs is closely correlated with poor prognosis in various cancers [62, 63]. However, the precise mechanisms through which GPNMB influences tumorigenesis in TAMs remain unclear. Our findings offer a foundation for future research in this area.

**Fig. 8:**
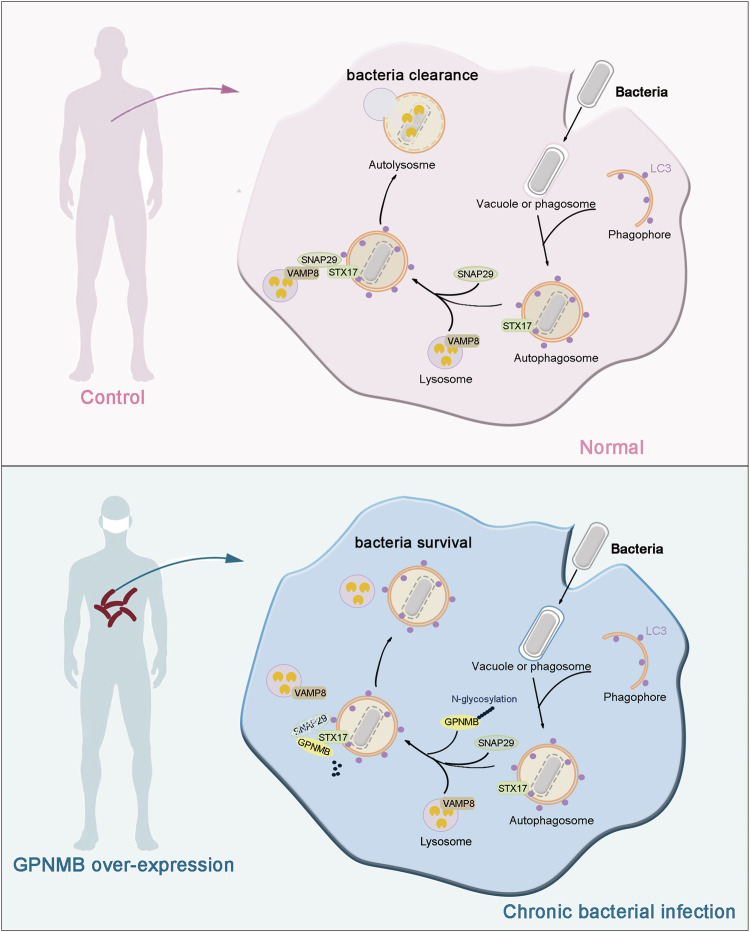
Model by which GPNMB maintains intracellular bacterial proliferation through blocking the assembly of the SNARE complex. During bacterial infection, GPNMB inhibits the fusion of autophagosomes with lysosomes by disrupting the assembly of the STX17-SNAP29-VAMP8 complex, thereby facilitating the replication of bacteria within macrophages. After bacterial infection, GPNMB binds to STX17, which promotes the deglycosylation of GPNMB and the degradation of SNAP29

In conclusion, our study revealed a negative role of GPNMB in regulating xenophagy upon bacterial infection (Fig. 8). GPNMB blocked the fusion of autophagosomes with lysosomes by disrupting the assembly of the STX17-SNAP29-VAMP8 SNARE complex. In addition to *M. leprae*, our findings demonstrated that GPNMB deficiency impaired the proliferation of other various intracellular bacteria in human macrophages. These findings highlight GPNMB as a potential therapeutic target for enhancing host immunity against intracellular bacterial infection, offering new avenues for the treatment of chronic infectious diseases.

## Materials and methods

### Animal studies

*Gpnmb*^fl/fl^ and *Gpnmb*^fl/fl^ Lyz2-Cre mice were purchased from GemPharmatech Co., Ltd. The genotyping of *Gpnmb*^fl/fl^ was confirmed via PCR via the following primers: 1 (5’ arm): forward 5’-AATGGGAGAGTAGGGTCCAGAGAG-3’, reverse 5’-GCCATCTCTCTAGTCCCCTCTCTT-3’; 2 (3’ arm): forward 5’-CACGGCTGT ATAGTGAGCCTTTCTC-3’, reverse 5’-CACTGGTGATCTTCCTGCCTCA-3’. The primers for genotyping Lyz2-Cre transgenic mice were as follows: forward, 5’-AGTGCTGAAG TCCATAGATCGG-3’; reverse, 1’-CTGATTCTCCTCATCAC C AGG-3’; and reverse, 2’-GTCACTCACTGCTCCCCTGT-3’. All mouse experiments were performed following the general guidelines published by the Association for Assessment and Accreditation of Laboratory Animal Care. All the mice were on the C57BL/6 background and were maintained under specific-pathogen-free conditions with the approval of the Scientific Investigation Board of the Shandong Provincial Hospital for Skin Diseases & Shandong Provincial Institute of Dermatology and Venereology.

### Cell lines

HEK293T and THP-1 cells were obtained from the American Type Culture Collection. *Gpnmb*-knockout THP-1 cells were purchased from Ubigene Biosciences. HEK293T cells were cultured at 37 °C under 5% CO_2_ in DMEM supplemented with 10% fetal bovine serum (FBS), 100 U/ml penicillin, and 100 µg/ml streptomycin. THP-1 cells and peripheral blood monocyte-derived macrophages (MDMs) were cultured at 37 °C under 5% CO_2_ in RPMI 1640 medium supplemented with 10% FBS, 100 U/ml penicillin and 100 µg/ml streptomycin. For the transfection of siRNA into cells, Lipofectamine™ RNAiMAX (Invitrogen, 13778150) was used. For the transfection of plasmids into HEK293T cells, Lipofectamine 3000 (Invitrogen, L3000015) was used. The siRNAs used in this study are shown in Supplementary Table S1. The plasmids used in this study are shown in Supplementary Table S2.

### Patients and clinical specimens

Patients (*n* = 15) with leprosy and healthy controls (*n* = 10) were recruited at Shandong Provincial Hospital for Skin Diseases (Jinan, China). Patients with leprosy were diagnosed according to previously described criteria [64]. Patients with leprosy were classified according to the criteria of Ridley and Jopling [65]. The designation of T-Lep included patients who were classified clinically as borderline tuberculoid (BT), and the designation of L-Lep included only patients classified as having lepromatous leprosy (LL). All T-lep and L-lep skin biopsy samples and sera were taken at the time of diagnosis, prior to initiating treatment. All skin samples and sera were stored at −80 °C before use.

### Microbes and reagents

The macrophages were infected with *M. leprae* (MOI of 10), *M. marinum* (MOI of 10), *S. typhimurium* (MOI of 1) *or L. monocytogenes* (MOI of 1) for the indicated times. To measure *M. marinum, S. typhimurium or L. monocytogenes* replication in macrophages, MDMs or THP-1 cells (90% confluent) in 12-well plates were infected with bacterial strains. After 1 h of incubation at 37 °C, the cells were washed three times with PBS to remove extracellular bacteria and incubated with fresh cell culture medium containing 100 μg/mL gentamycin. Four hours (for *S. typhimurium or L. monocytogenes* infection) or 24 h (for *M. marinum* infection) later, the cells were lysed in cold PBS containing 0.1% Triton X-100, and the number of colony-forming units was determined via serial dilution plating on Middlebrook 7H9 (for *M. marinum* infection), LB (for *S. typhimurium* infection) or Brain Heart Infusion (BHI, for *L. monocytogenes* infection) plates. To measure the survival rate of *M. leprae*, DNA/RNA was extracted via the Quick-DNA/RNA^TM^ Pathogen MagBead (ZOMO RESEARCH, R2145). *M. leprae* viability was determined via qPCR and quantified [66].

Reagents, including M-CSF (proteintech, HZ-1192), IFN-γ (R&D Systems, 285-IF-100/CF), rapamycin (MCE, AY-22989), Torin 1 (Selleck, S2827), bafilomycin A1 (MCE, HY-100558), CQ (MCE, HY-17589A) and CHX (MCE, HY-12320), were used at the indicated concentrations (shown in the figure legends).

### RNA quantification

Total RNA was extracted via an RNA fast200 kit (Fastagen, 220011) and reverse transcribed via reverse transcriptase (Takara, RR047A). The primers used for the RT‒PCR assays are shown in Supplementary Table S3. Quantitative real-time PCR analysis was performed on a StepOnePlus real-time PCR system (Applied Biosystems) using FastStart Universal SYBR Green Master (Rox) (Roche, 4913850001). The mRNA levels of the genes were normalized to the expression of *β-actin* in each individual sample. The 2^−ΔΔCt^ method was used to calculate relative expression changes.

### ELISA

The concentrations of IL-6 (1110602), TNF-α (1117202), and IL-1β (1110122) in the culture supernatants were measured via ELISA kits (DAKEWE). The concentrations of IL-6 (1210602), TNF-α (1217202), IL-1β (1210122), IL-4 (1210402), IL-10 (1211002) and IFN-γ (1210002) in mouse tissues were measured via ELISA kits (DAKEWE) according to the manufacturer’s directions. Patient sera were collected, and the concentration of GPNMB was measured via human osteoactivin/GPNMB DuoSet ELISA (R&D System, DY2550).

### Phagocytosis

Phagocytosis assays were performed by using a Phagocytosis Assay Kit (IgG FITC) (Cayman, 500290) according to the manufacturer’s protocol.

### Monocyte isolation

Human peripheral blood mononuclear cells (PBMCs) were prepared by using a human lymphocyte separation tube (DAKEWE, 7121011), followed by CD14^+^ monocyte isolation with CD14 microbeads (Miltenyi Biotec, 130-050-201) according to the manufacturer’s protocol. The number of cells was determined, and the cells were plated in dishes. The cells were cultured with RPMI 1640 + 10% FBS + 100 U/ml penicillin + 100 µg/ml streptomycin + M-CSF (50 ng/mL) for up to 7 days.

### Coimmunoprecipitation (Co-IP) and immunoblot analysis

For immunoprecipitation (IP), whole-cell extracts were collected and lysed in IP buffer containing 1% (vol/vol) NP-40, 50 mM Tris-HCl (pH 7.4), 50 mM EDTA, 150 mM NaCl, and EDTA-free protease inhibitor cocktail (Roche, 4693132001). After centrifugation for 10 min at 14,000 × *g*, the supernatants were collected and incubated with specific antibodies at 4 °C for 2 h, after which protein A/G magnetic beads for IP (SELLECK, B23202) were added. After incubation overnight, the beads were washed five times with 1× TBST buffer (Solarbio, T1082). Immunoprecipitates were eluted by boiling with 1× protein loading buffer (TransGen Biotech, DL101-02) for 10 min. For immunoblot analysis, immunoprecipitates or whole-cell lysates were loaded and subjected to SDS–PAGE, transferred onto nitrocellulose membranes and then blotted with specific antibodies. The antibodies used in this study are shown in Supplementary Table S4.

### Immunofluorescence staining and microscopy

The cells were fixed for 10 min with 4% PFA and permeabilized for 10 min at room temperature with 0.5% Triton X-100. After blocking nonspecific binding for 1 h, primary antibodies were added, and the samples were incubated for 2 h at room temperature. The samples were further stained with suitable Alexa Fluor 488-, Alexa Fluor 568-, or Alexa Fluor 647-conjugated secondary antibodies (Thermo Fisher Scientific). Nuclei were stained with 4’,6’-diamidino-2-phenylindole dihydrochloride (DAPI, Abcam). Images were acquired via confocal laser microscopy (LSM900, Carl Zeiss). The antibodies used in this study are shown in Supplementary Table S4.

### M. leprae labeling

*M. leprae* was grown on the footpads of nu/nu mice and isolated as described previously [67]. Live *M. leprae* were labeled with a PKH26 Red Fluorescent Cell Linker Mini Kit for General Cell Membrane Labeling (Sigma-Aldrich, MINI26-1KT) according to the manufacturer’s protocol.

### Mouse model of M. marinum infection

*M. marinum* strains were cultured at 30 °C in 20 ml of Mycobacterium Complete Medium (Gene Optimal, GOMY0026). Several days later, the cultures were counted via a hemocytometer. The *M. marinum* strains were injected via the lateral tail vein with 2 × 10^7^ CFUs (colony forming units). The mice were monitored closely for 21 days for signs of illness. For the bacterial loading experiment, the mice were sacrificed at 7, 14, and 21 days post infection. The tails, spleens and livers were harvested, homogenized, serially diluted in PBS, and plated on Middlebrook 7H10 solid culture medium (HOPEBIO, HBPM6270). The plates were incubated at 30 °C for 7 days to determine the CFU per gram of tissue. For immunohistochemistry analysis, tails were removed from the mice, and a clean razor used to slice tails into 3–5 mm lengths that were placed in a cassette and submerged in 10% neutral buffered formalin for 24 h. Tail sections were washed and transferred to JYBL-I Decalcifying Solution (Solarbio, G2470) for 24 h before being moved to 70% ethanol for storage until the sections were embedded in paraffin and sectioned. Sections (thickness = 2 μm) were subjected to H&E staining. For soluble protein isolation for ELISA, 3–5 mm sections of the tail were cut fresh from the tail and immediately placed in liquid nitrogen. The samples were stored at −80 °C until processing. The samples were weighed and placed in PBS, and scissors were used to separate the tissue before it was homogenized in a gentleMACS Dissociator (Miltenyi Biotec). The concentrations of IL-6, TNF-α, IL-1β, IL-4, IL-10 and IFN-γ were measured via ELISA kits (DAKEWE) according to the manufacturer’s directions. For RNA quantification, 3–5 mm sections of the tail were cut fresh from the areas containing granulomatous lesions or near the injection site for uninfected mice or mice with no visible lesions and immediately placed in TRIzol (Invitrogen). The tissue was cut with sterile scissors and then homogenized in a gentleMACS Dissociator (Miltenyi Biotec). RNA extraction was performed in accordance with the manufacturer’s protocols.

### Lysosomal pH measurement

HEK293T cells were seeded on coverslips and incubated at 37 °C under 5% CO_2_ overnight. The culture medium was removed, and the LysoSensor™ yellow/blue DND-160 probe (1 μM, Thermo Fisher, L7545)-containing medium was added. The cells were incubated at 37 °C under 5% CO_2_ for 5 min. The loading mixture was then replaced with fresh medium, and the cells were observed via confocal laser microscopy (LSM900, Carl Zeiss).

### Lysosomal intracellular activity assay

The assay was performed by using a lysosomal intracellular activity assay kit (Abcam, ab234622). HEK293T cells or THP-1 cells were seeded on coverslips and incubated at 37 °C under 5% CO_2_ overnight. The media was removed, culture medium supplemented with 0.5% FBS was added, and the cells were incubated at 37 °C under 5% CO_2_ for 1 h. The culture media was then changed to self-quenched-containing medium supplemented with 0.5% FBS for an additional 1 h according to the kit’s protocol. Images were taken via confocal laser microscopy (LSM900, Carl Zeiss).

### Quantification and statistical analysis

All the data are presented as the means ± SDs of three or more experiments. Statistical significance was determined with a two-tailed Student’s *t* test, with *p* < 0.05 considered statistically significant. The *p* values are represented as *p < 0.05, **p < 0.01, ***p < 0.001, and ****p < 0.0001. We performed the statistical analyses by using GraphPad Prism 8. Statistical details are shown in each of the figure legends. Protein expression levels were quantitated by measuring band intensities via ImageJ software. No data points or mice were excluded from the study. No randomization or blinding was used.

## Supplementary information

Supplementary-Tables and figures

Supplementary-original images of gels
