# Supplementary material for: GPNMB disrupts SNARE complex assembly to maintain bacterial proliferation within macrophages
Source: Cell Mol Immunol. 2025 Mar 4;22(5):512–26. doi: 10.1038/s41423-025-01272-z (PMC12041529; doi:10.1038/s41423-025-01272-z)
Supplement: Supplementary file 1 — Supplementary-Tables and figures [file 41423_2025_1272_MOESM1_ESM.pdf]

Supplemental information

Figures and tables

GPNMB disrupts SNARE complex assembly to maintain bacteria proliferation within macrophages

RUNNING TITLE: The effect of GPNMB upon bacterial infection

Zhenzhen Yan[1,2,3], Jinghong Han[1,2], Zihao Mi[1,2], Zhenzhen Wang[1,2], Yixuan Fu[3], Chuan Wang[1,2], Ningning Dang[3], Hong Liu[1,2,4,*], Furen Zhang[1,2,4,*]

[1]Hospital for Skin Diseases, Shandong First Medical University, Jinan, Shandong, China

[2]Shandong Provincial Institute of Dermatology and Venereology, Shandong Academy of Medical Sciences, Jinan, Shandong, China

[3]Department of Dermatology, Shandong Provincial Hospital Affiliated to Shandong First Medical University, Jinan, Shandong, China

[4]School of Public Health, Shandong First Medical University & Shandong Academy of Medical Sciences, Jinan, Shandong, China

*Correspondence: hongyue2519@hotmail.com (H.L.), zhangfuren@hotmail.com (F.Z.)

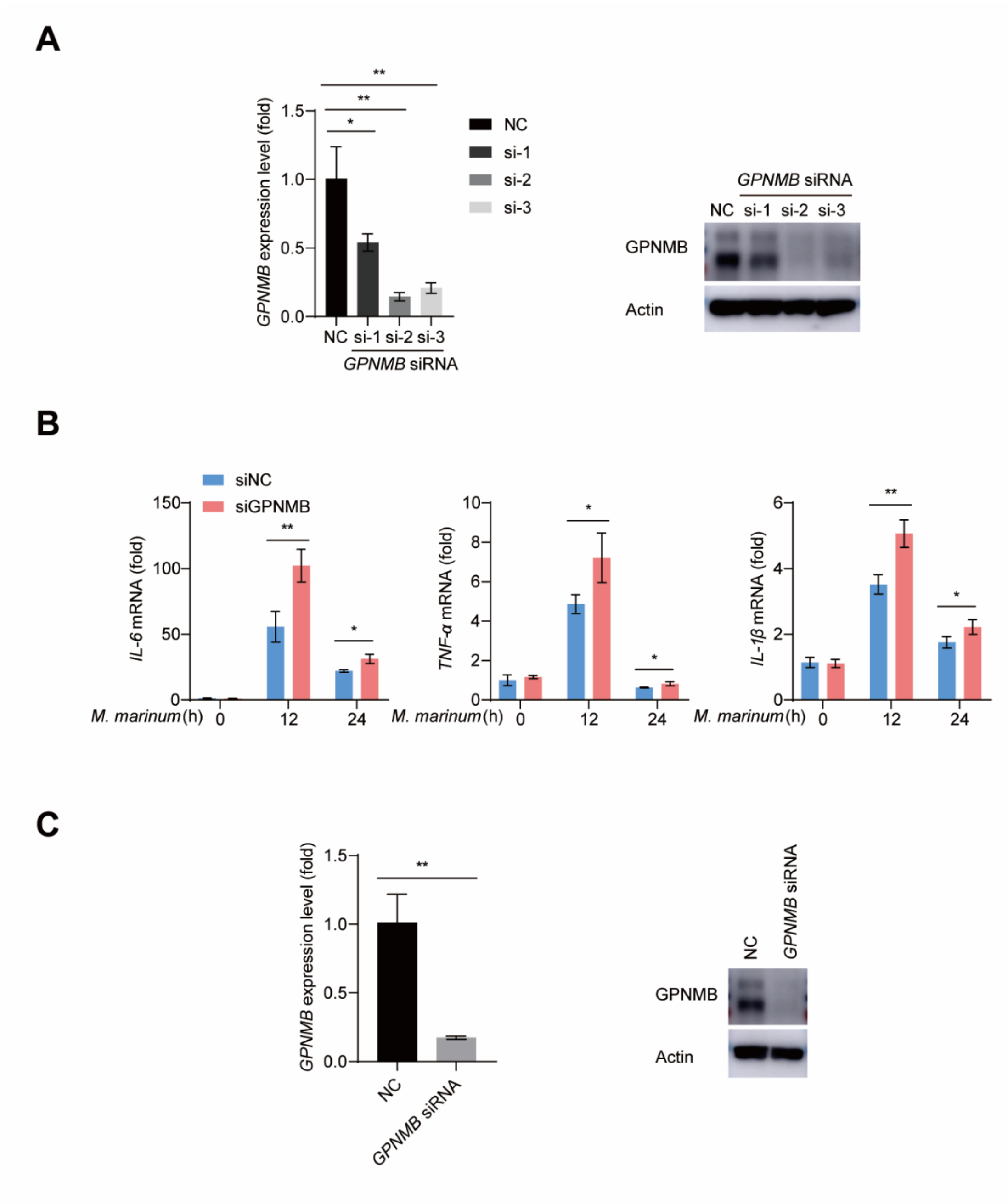

Figure S1. GPNMB negatively regulates pro-inflammatory cytokines production. (**A**) Knockdown efficiency of *GPNMB* in THP-1 cells. (**B**) qPCR analysis of *IL-6*, *TNF-α*, and *IL-1β* mRNA level in siNC or siGPNMB transfected THP-1 cells infected with *M. marinum* (10 MOI) for indicated times, n=3. (**C**) Knockdown efficiency of *GPNMB* in MDMs. Data are shown as mean ± SD and were analyzed by unpaired two tailed *t* test (*p<0.05, **p<0.01).

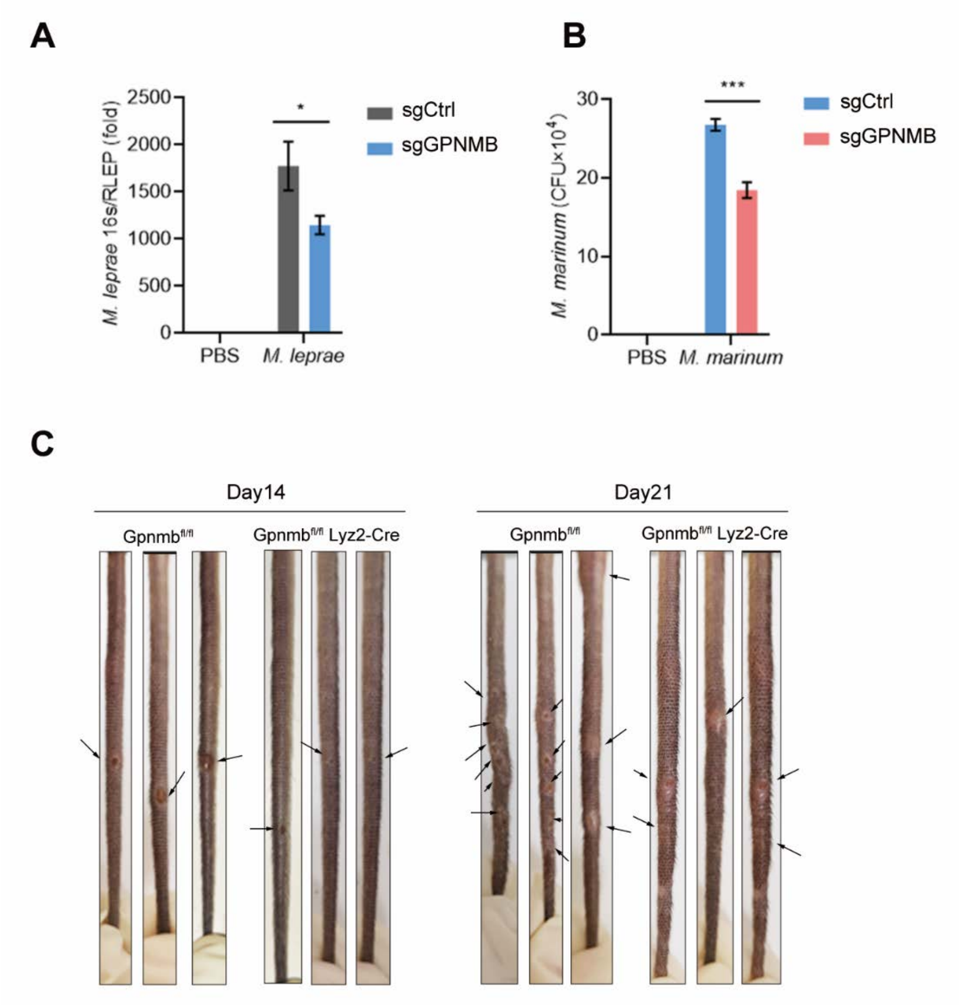

Figure S2. GPNMB negatively regulates the antibacterial immunity.

(**A**) sgCtrl and *Gpnmb*-knockout THP-1 cells were infected with *M. leprae* (10 MOI) for 24 h. Viability of *M. leprae* was calculated by the ratio of bacterial 16S rRNA and DNA (RLEP) detected by qPCR. (**B**) sgCtrl and *Gpnmb*-knockout THP-1 cells were infected with *M. marinum* (10 MOI) for 24 h. The intracellular amounts of bacteria were quantified. (**C**) *Gpnmb*$^{fl/fl}$ and *Gpnmb*$^{fl/fl}$ Lyz2-Cre mice were infected with *M. marinum* ($2x10^7$ CFU per mouse). Representative images of tail lesions were shown at indicated times post infection. Data are shown as mean ± SD and were analyzed by unpaired two tailed *t* test (*p<0.05, ***p<0.001).

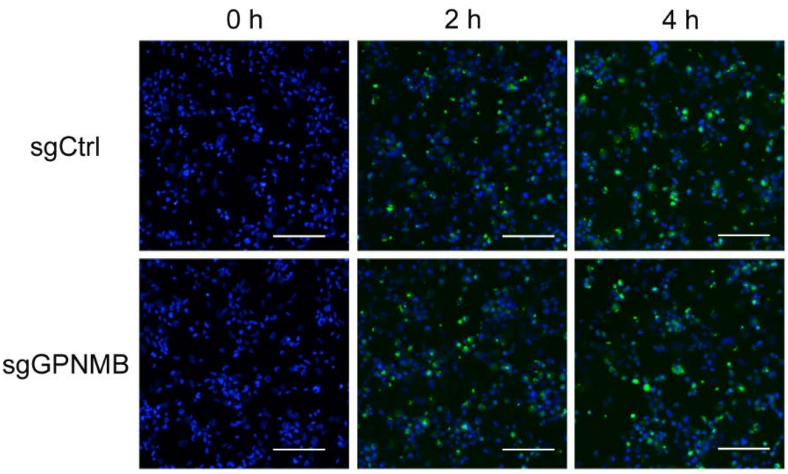

Figure S3. The phagocytosis of THP-1 cells cannot be triggered by GPNMB.

sgCtrl and *Gpnmb*-knockout THP-1 cells were incubated with Latex beads-rabbit IgG-FITC complex for 0-4 h. The phagocytic activity of THP-1 cells was measured by microscopy images. Scale bar, 100 μm.

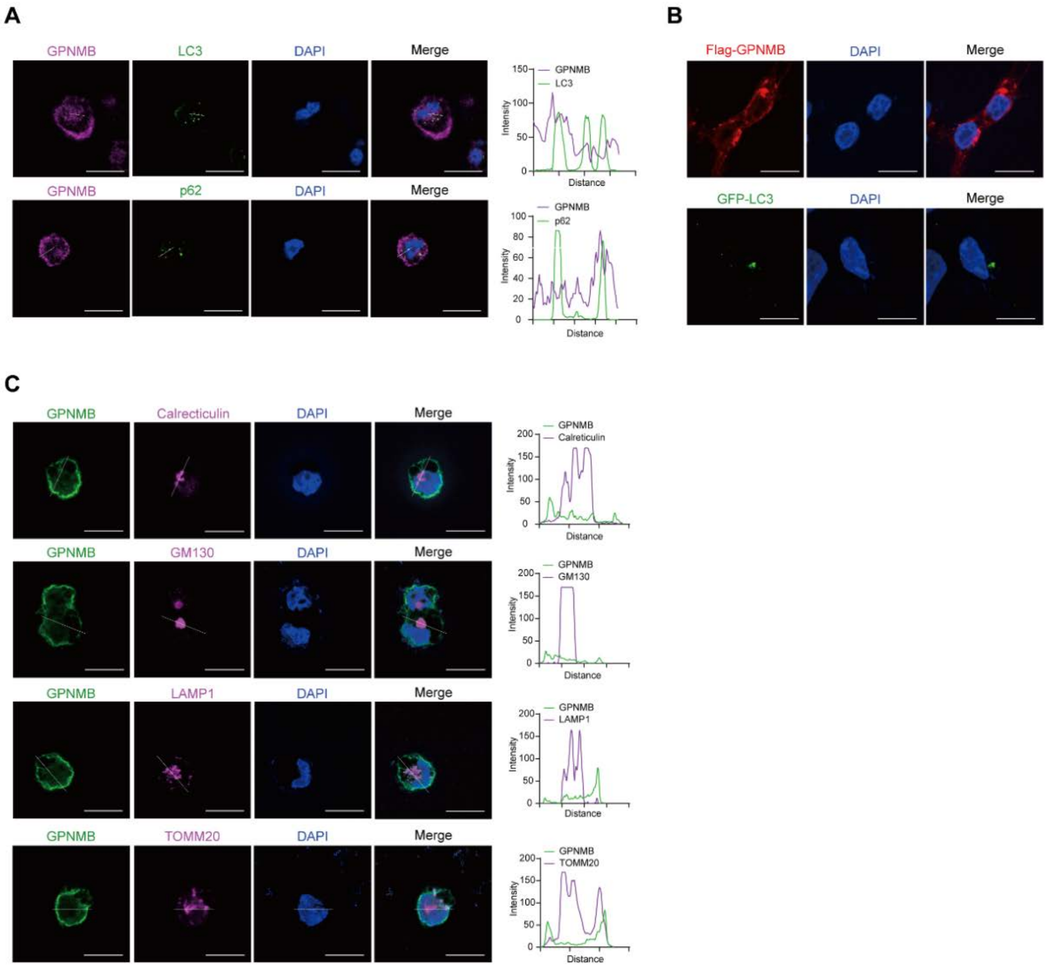

Figure S4. The localization of GPNMB in uninfected cells.

(**A**) Confocal microscopy of THP-1 cells. Cells were stained with antibodies labeling GPNMB (purple), LC3 (green), p62 (green) and the DNA-binding dye DAPI (blue). Scale bar, 10 μm. (**B**) Confocal microscopy of HEK293T cells transfected with Flag-GPNMB or GFP-LC3 for 24 h. Cells were stained with antibodies labeling Flag (red) and the DNA-binding dye DAPI (blue). Scale bar, 10 μm. (**C**) Confocal microscopy of THP-1 cells. Cells were stained with antibodies labeling GPNMB (green), Calreticulin (ER marker, purple), GM130 (Golgi marker, purple), LAMP1 (lysosome marker, purple), TOMM20 (mitochondria marker, purple) and the DNA-binding dye DAPI (blue). Scale bar, 10 μm.

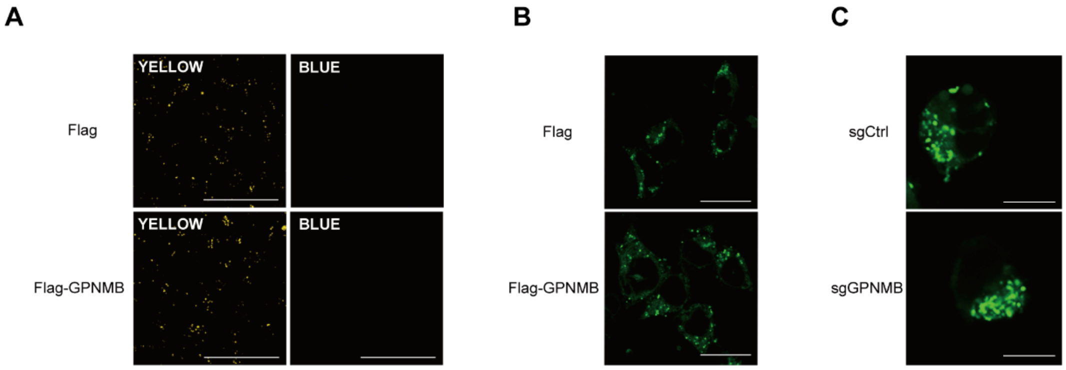

Figure S5. GPNMB has no effect on the acidic acidic environment or the intracellular activity of lysosome.

(**A**) Confocal microscopy of HEK293T cells transfected with control or Flag-GPNMB expression plasmid for 24 h. Cells were treated with 1 μM LysoSensor™ Yellow/Blue DND-160 probe for 5 min. Scale bar, 50 μm. (**B**) Confocal microscopy of HEK293T cells transfected with control or Flag-GPNMB expression plasmid for 24 h. Cells were treated with Lysosome-Specic Self-Quenched Substrate for 1 h according to kit's protocol. Scale bar, 20 μm. (**C**) Confocal microscopy of sgCtrl or sgGPNMB THP-1 cells. Cells were treated with Lysosome-Specic Self-Quenched Substrate for 1 h according to kit's protocol. Scale bar, 10 μm.

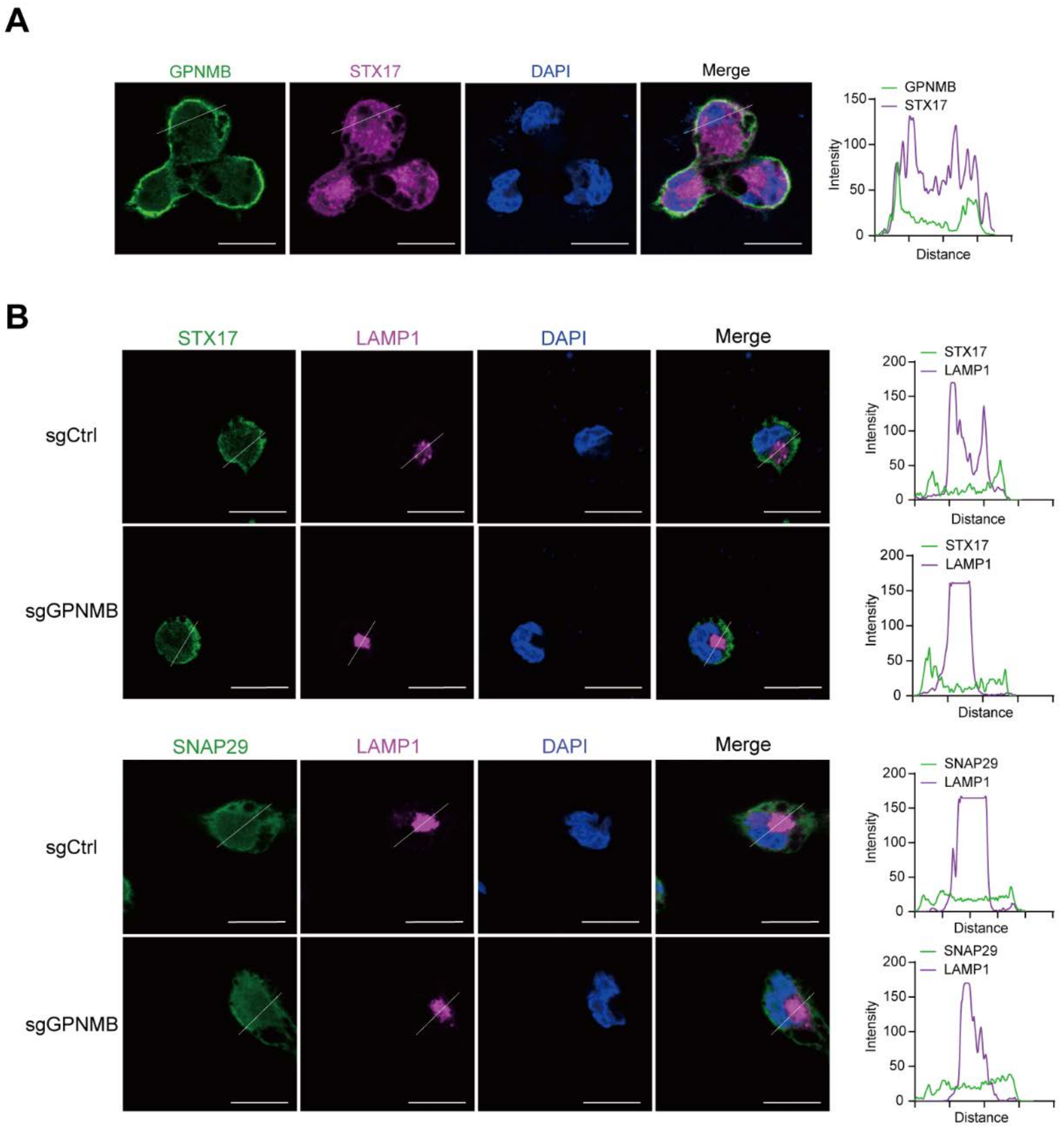

Figure S6. The localization of STX17 and SNAP29 in uninfected cells.

(**A**) Confocal microscopy of THP-1 cells. Cells were stained with antibodies labeling GPNMB (green), STX17 (purple) and the DNA-binding dye DAPI (blue). Scale bar, 10 μm. (**B**) Confocal microscopy of sgCtrl and sgGPNMB THP-1 cells. Cells were stained with antibodies labeling STX17 (green), SNAP29 (green), LAMP1 (purple) and the DNA-binding dye DAPI (blue). Scale bar, 10 μm.

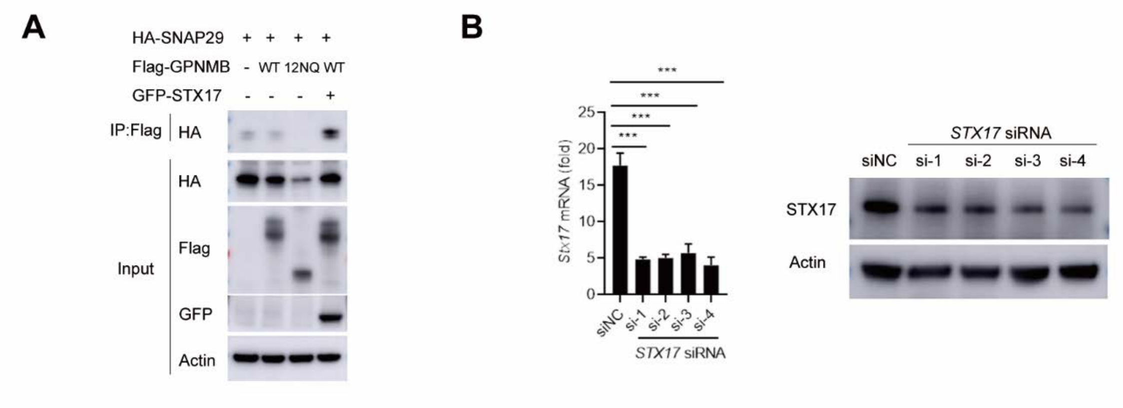

Figure S7. SNAP29 binds with GPNMB-STX17 complex.

(**A**) HEK293T cells were transfected with HA-SNAP29, Flag-GPNMB (WT) or Flag-GPNMB (12NQ), in the present of GFP-STX17 expression plasmid or not for 24 h. Co-IP and immunoblots were performed with the indicated antibodies. (**B**) Knockdown efficiency of STX17 in THP-1 cells. Data are shown as mean ± SD and were analyzed by unpaired two tailed *t* test (***p<0.001).

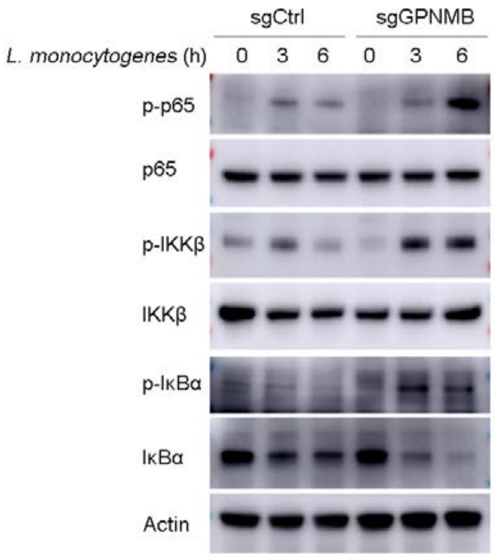

Figure S8. GPNMB inhibits the activation of NF-κB signaling pathway.

sgCtrl and *Gpnmb*-knockout THP-1 cells were infected with *L. monocytogenes* (1 MOI) for indicated times. The phosphorylation level of p65, IKKβ and IκBα was measured by Western blot.

Table S1. Oligonucleotides used in this study.

Oligonucleotide	Forward primer (5'-3')	Reverse primer (5'-3')
GPNMB-Homo-1	GUGGGCUCAAAUAUAACAUTT	AUGUUAUAUUUGAGCCCACTT
GPNMB-Homo-2	CUAGCCACUUCCUCAAUUATT	UAAUUGAGGAAGUGGCUAGTT
GPNMB-Homo-3	GCUCCCUAAUAGACUUUGUTT	ACAAAGUCUAUUAGGGAGCTT
STX17-Homo-1	GAACCAGCUAUCCAGAAAUTT	AUUUCUGGAUAGCUGGUUCTT
STX17-Homo-2	GAUCCAAUAUCCGAGAAAUTT	AUUUCUCGGAUAUUGGAUCTT
STX17-Homo-3	GCGGACUUAAUUGAACUUATT	UAAGUUCAAUUAAGUCCGCTT
STX17-Homo-4	CAGCCAAACUGACAAGAAATT	UUUCUUGUCAGUUUGGCUGTT

Table S2. Plasmids used in this study.

Plasmid	Source	Identifier
pCMV-GPNMB(human)-3×FLAG-Neo	MIAOLING BIOLOGY	Cat# P44030
pCMV-3×Myc-VAMP8(human)-Neo	MIAOLING BIOLOGY	Cat# P45078
pCMV-3×HA-SNAP29(human)-Neo	MIAOLING BIOLOGY	Cat# P45119
pEnCMV-EGFP-Linker-STX7(human)-SV40-Neo(human)	MIAOLING BIOLOGY	Cat# P26659
pCDNA3.1-STX17(human)-Linker-EGFP-SV40-Neo	MIAOLING BIOLOGY	Cat# P24326
pCDNA3.1-STX17(1-149)(human)-Linker-EGFP-SV40-Neo	This Study	N/A
pCDNA3.1-STX17(1-224)(human)-Linker-EGFP-SV40-Neo	This Study	N/A
pCDNA3.1-STX17(150-302)(human)-Linker-EGFP-SV40-Neo	This Study	N/A
pCDNA3.1-STX17(276-302)(human)-Linker-EGFP-SV40-Neo	This Study	N/A
pCDNA3.1-STX17(Δ162-224)(human)-Linker-EGFP-SV40-Neo	This Study	N/A
pCDNA3.1-STX17(229-275)(human)-Linker-EGFP-SV40-Neo	This Study	N/A
pCMV-YKT6-3×Myc-Neo	MIAOLING BIOLOGY	Cat# P52322
pCMV3-HA-ULK1(human)	MIAOLING BIOLOGY	Cat# P5225
pCMV-Myc-BECN1	MIAOLING BIOLOGY	Cat# P6909
pCMV-GPNMB(12NQ)(human)-3×FLAG-Neo	This Study	N/A
pCMV-GPNMB(93Q)(human)-3×FLAG-Neo	This Study	N/A
pCMV-GPNMB(134Q)(human)-3×FLAG-Neo	This Study	N/A
pCMV-GPNMB(146Q)(human)-3×FLAG-Neo	This Study	N/A
pCMV-GPNMB(200Q)(human)-3×FLAG-Neo	This Study	N/A

pCMV-GPNMB(249Q)(human)-3×FLAG-Neo	This Study	N/A
pCMV-GPNMB(275Q)(human)-3×FLAG-Neo	This Study	N/A
pCMV-GPNMB(296Q)(human)-3×FLAG-Neo	This Study	N/A
pCMV-GPNMB(300Q)(human)-3×FLAG-Neo	This Study	N/A
pCMV-GPNMB(306Q)(human)-3×FLAG-Neo	This Study	N/A
pCMV-GPNMB(312Q)(human)-3×FLAG-Neo	This Study	N/A
pCMV-GPNMB(459Q)(human)-3×FLAG-Neo	This Study	N/A
pCMV-GPNMB(467Q)(human)-3×FLAG-Neo	This Study	N/A
pCMV-GFP-LC3B	Beyotime	Cat# D2815-1μg
pCMV-mCherry-GFP-LC3B	Beyotime	Cat# D2816-1μg

Table S3. Primers used in this study.

Gene	Species	Forward primer (5’-3’)	Reverse primer (5’-3’)
GPNMB	Human	AACCTTGAGTGCCTGC GTCC	AGGGCTGGTGAGTCAC TGGT
IL-6	Human	AGACAGCCACTCACCT CTTCAG	TTCTGCCAGTGCCTCT TTGCTG
TNF-α	Human	CTCTTCTGCCTGCTGC ACTTTG	ATGGGCTACAGGCTTG TCACTC
IL-1β	Human	TGATGGCTTATTACAGT GGCA	GGTCGGAGATTCGTAG CTGG
ACTIN	Human	GGAAATCGTGCGTGAC ATTAA	AGGAAGGAAGGCTGG AAGAG
Il-6	Mouse	ACAACCACGGCCTTCC CTAC	CATTTCCACGATTTCCC AGA
Tnf-α	Mouse	GCCACCACGCTCTTCT GTCT	TGAGGGTCTGGGCCAT AGAAC
Il-1β	Mouse	ACCTTCCAGGATGAGG ACATGA	AACGTCACACACCAGC AGGTTA
Il-4	Mouse	ATCATCGGCATTTTGA ACGAGGTC	ACCTTGGAAGCCCTAC AGACGA
Il-10	Mouse	CGGGAAGACAATAACT GCACCC	CGGTTAGCAGTATGTT GTCCAGC
Ifn-γ	Mouse	CAGCAACAGCAAGGC GAAAAAGG	TTTCCGCTTCCTGAGG CTGGAT
Actin	Mouse	CCACACCCGCCACCAG TTCG	TACAGCCCGGGGAGC ATCGT
M. leprae 16s rRNA		GCATGTCTTGTGGTGG AAAGC	CACCCCACCAACAAGC TGAT
M. leprae RLEP DNA		GCAGCAGTATCGTGTT AGTGAA	CGCTAGAAGGTTGCCG TAT

Table S4. Antibodies used in this study.

Antibody	Source	Identifier
For Co-IP and immunoblot analysis		
LC3B (D11) XP® Rabbit mAb	Cell Signaling Technology	3868S
GPNMB (E4D7P) XP® Rabbit mAb	Cell Signaling Technology	38313S
LAMP1 (D2D11) XP® Rabbit mAb	Cell Signaling Technology	9091S
Normal Rabbit IgG	Cell Signaling Technology	2729S
SQSTM1/p62 (D1Q5S) Rabbit mAb	Cell Signaling Technology	39749S
Phospho-Beclin-1 (Ser93) (D9A5G) Rabbit mAb	Cell Signaling Technology	4717S
Phospho-ULK1 (Ser317) Antibody	Cell Signaling Technology	37762S
Phospho-ULK1 (Ser555) (D1H4) Rabbit mAb	Cell Signaling Technology	5869S
DYKDDDDK Tag (D6W5B) Rabbit mAb	Cell Signaling Technology	14793S
HA-Tag (6E2) Mouse mAb	Cell Signaling Technology	2367S
Syntaxin 17 (D3D7H) Rabbit mAb	Cell Signaling Technology	31261S
GFP Monoclonal Antibody (GF28R)	Thermofisher	MA5-15256
9E10, Anti-Myc Monoclonal Antibody	origene	TA150121
N-Acetylglucosamine (GlcNAc) Mouse Monoclonal Antibody	origene	AM00063PU-N
DYKDDDDK Tag Monoclonal Antibody (FG4R)	Invitrogen	MA1-91878
SNAP29 Polyclonal antibody	proteintech	12704-1-AP
Monoclonal ANTI-FLAG® M2 antibody produced in mouse	sigma	F1804
Anti-Actin Antibody	ZSGB-BIO	ZM-0001
For Immunofluorescence staining		
LC3B (D11) XP® Rabbit mAb	Cell Signaling Technology	3868S
LC3B (E5Q2K) Mouse mAb	Cell Signaling Technology	83506

SQSTM1/p62 (D6M5X) Rabbit mAb	Cell Signaling Technology	23214
Recombinant Anti-GM130 antibody	abcam	ab52649
Recombinant Anti-Calreticulin antibody	abcam	ab92516
Recombinant Anti-TOMM20 antibody	abcam	ab186735
Anti-Listeria monocytogenes antibody	abcam	ab35132
GPNMB Antibody (D-9)	Santa Cruz Biotechnology	sc-271415
Syntaxin 17 Antibody (A-11)	Santa Cruz Biotechnology	sc-518187
SNAP 29 Antibody (D-8)	Santa Cruz Biotechnology	sc-390801
DYKDDDDK Tag Monoclonal Antibody (FG4R)	Invitrogen	MA1-91878
SNAP29 Polyclonal Antibody	Invitrogen	PA5-78285
Goat anti-Rabbit IgG (H+L) Highly Cross-Adsorbed Secondary Antibody, Alexa Fluor™ 488	Thermofisher	A-11034
Goat anti-Rabbit IgG (H+L) Cross-Adsorbed Secondary Antibody, Alexa Fluor™ 568	Thermofisher	A-11011
Goat anti-Mouse IgG (H+L) Cross-Adsorbed Secondary Antibody, Alexa Fluor™ 488	Thermofisher	A-11001
Donkey anti-Mouse IgG (H+L) Highly Cross-Adsorbed Secondary Antibody, Alexa Fluor™ 568	Thermofisher	A10037
Goat anti-Rabbit IgG (H+L) Highly Cross-Adsorbed Secondary Antibody, Alexa Fluor™ Plus 647	Thermofisher	A32733
