# Supplementary material for: GPNMB disrupts SNARE complex assembly to maintain bacterial proliferation within macrophages
Source: Cell Mol Immunol. 2025 Mar 4;22(5):512–26. doi: 10.1038/s41423-025-01272-z (PMC12041529; doi:10.1038/s41423-025-01272-z)

Supplemental information

Original images of gels

GPNMB disrupts SNARE complex assembly to maintain bacteria proliferation within macrophages

RUNNING TITLE: The effect of GPNMB upon bacterial infection

Zhenzhen Yan[1,2,3], Jinghong Han[1,2], Zihao Mi[1,2], Zhenzhen Wang[1,2], Yixuan Fu[3], Chuan Wang[1,2], Ningning Dang[3], Hong Liu[1,2,4,*], Furen Zhang[1,2,4,*]

[1]Hospital for Skin Diseases, Shandong First Medical University, Jinan, Shandong, China

[2]Shandong Provincial Institute of Dermatology and Venereology, Shandong Academy of Medical Sciences, Jinan, Shandong, China

[3]Department of Dermatology, Shandong Provincial Hospital Affiliated to Shandong First Medical University, Jinan, Shandong, China

[4]School of Public Health, Shandong First Medical University & Shandong Academy of Medical Sciences, Jinan, Shandong, China

*Correspondence: hongyue2519@hotmail.com (H.L.), zhangfuren@hotmail.com (F.Z.)

Fig. 3C

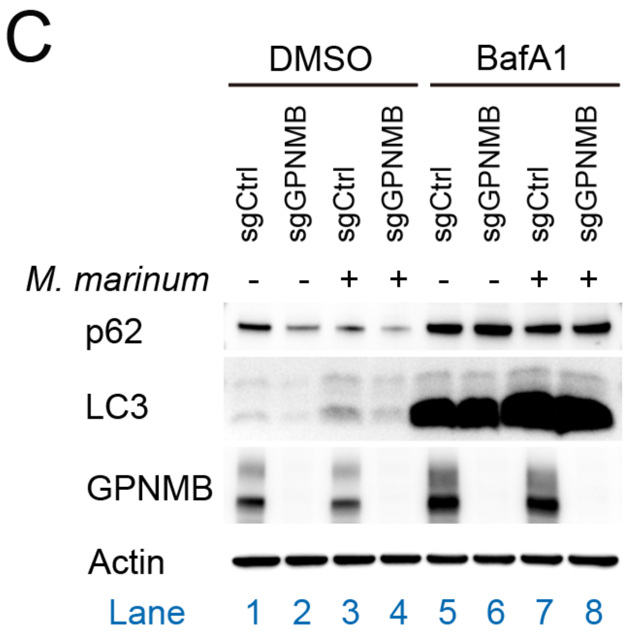
C
DMSO
BafA1
sgCtrl
sgGPNMB
sgCtrl
sgGPNMB
sgCtrl
sgGPNMB
sgCtrl
sgGPNMB
M. marinum
- - + + - - + +
p62
LC3
GPNMB
Actin
Lane
1 2 3 4 5 6 7 8

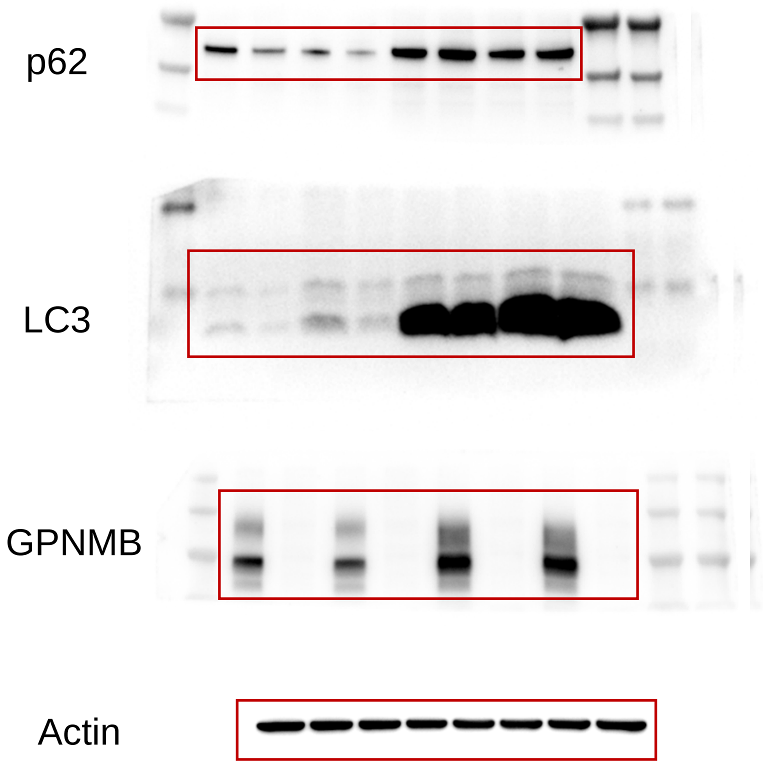
p62
LC3
GPNMB
Actin

Fig. 3E

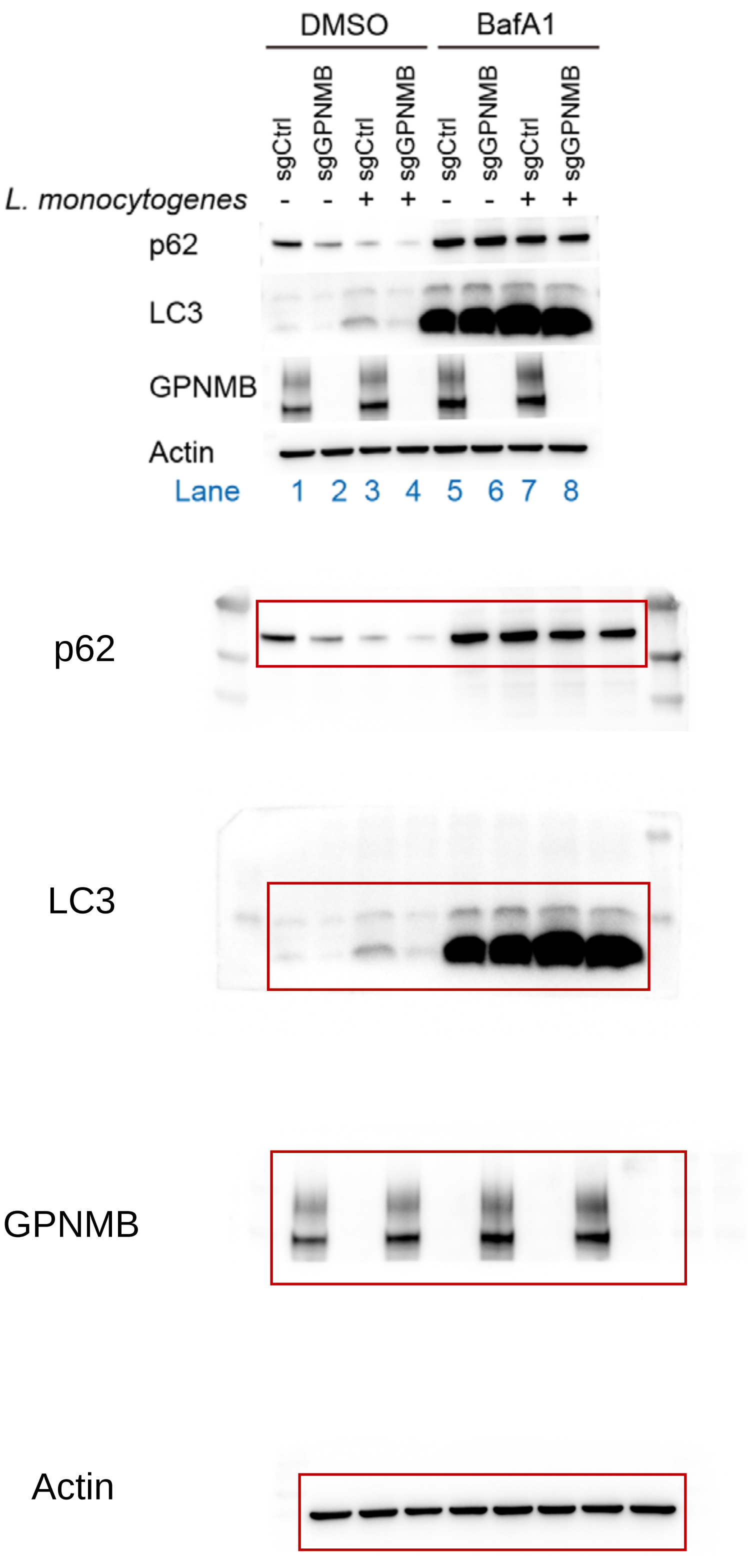
DMSO
BafA1
sgCtrl
sgGPNMB
sgCtrl
sgGPNMB
sgCtrl
sgGPNMB
sgCtrl
sgGPNMB
L. monocytogenes
- - + + - - + +
p62
LC3
GPNMB
Actin
Lane
1 2 3 4 5 6 7 8
p62
LC3
GPNMB
Actin

Fig. 3H

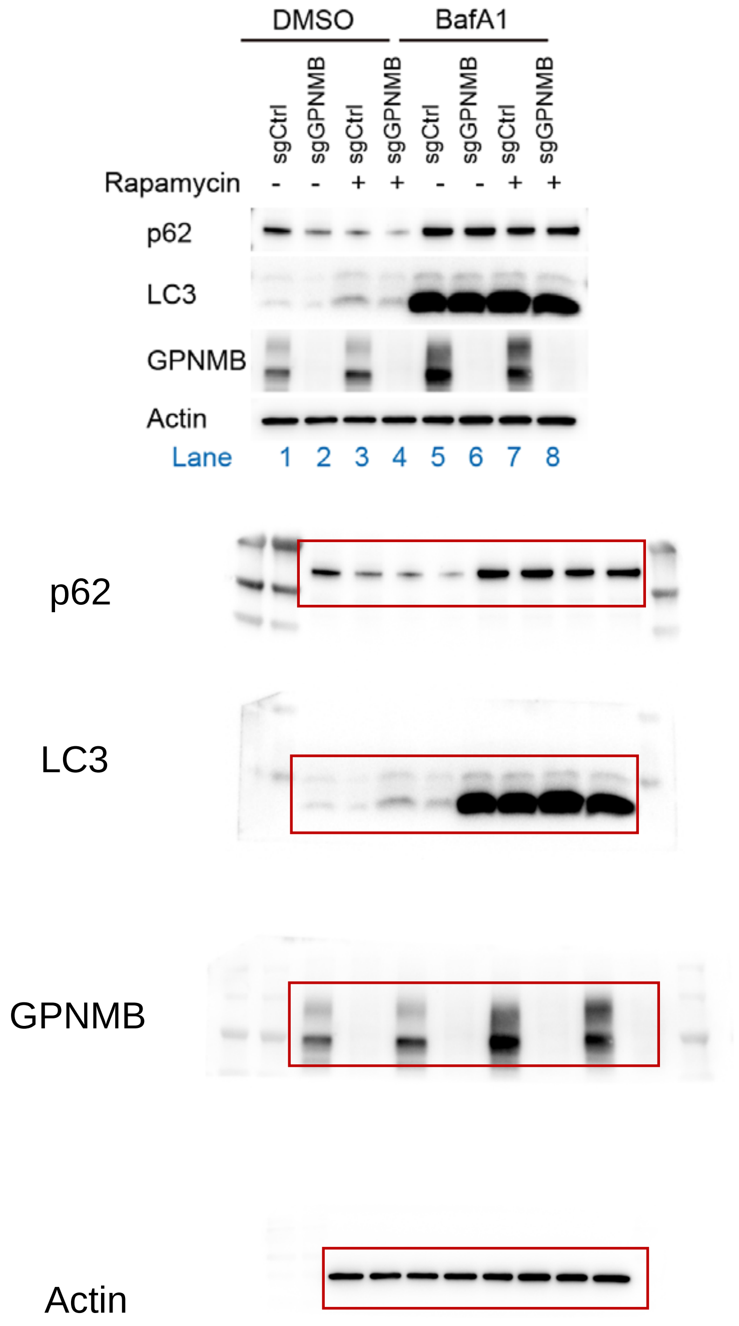
DMSO
BafA1
sgCtrl
sgGPNMB
sgCtrl
sgGPNMB
sgCtrl
sgGPNMB
sgCtrl
sgGPNMB
Rapamycin - - + + - - + +
p62
LC3
GPNMB
Actin
Lane 1 2 3 4 5 6 7 8
p62
LC3
GPNMB
Actin

Fig. 3J

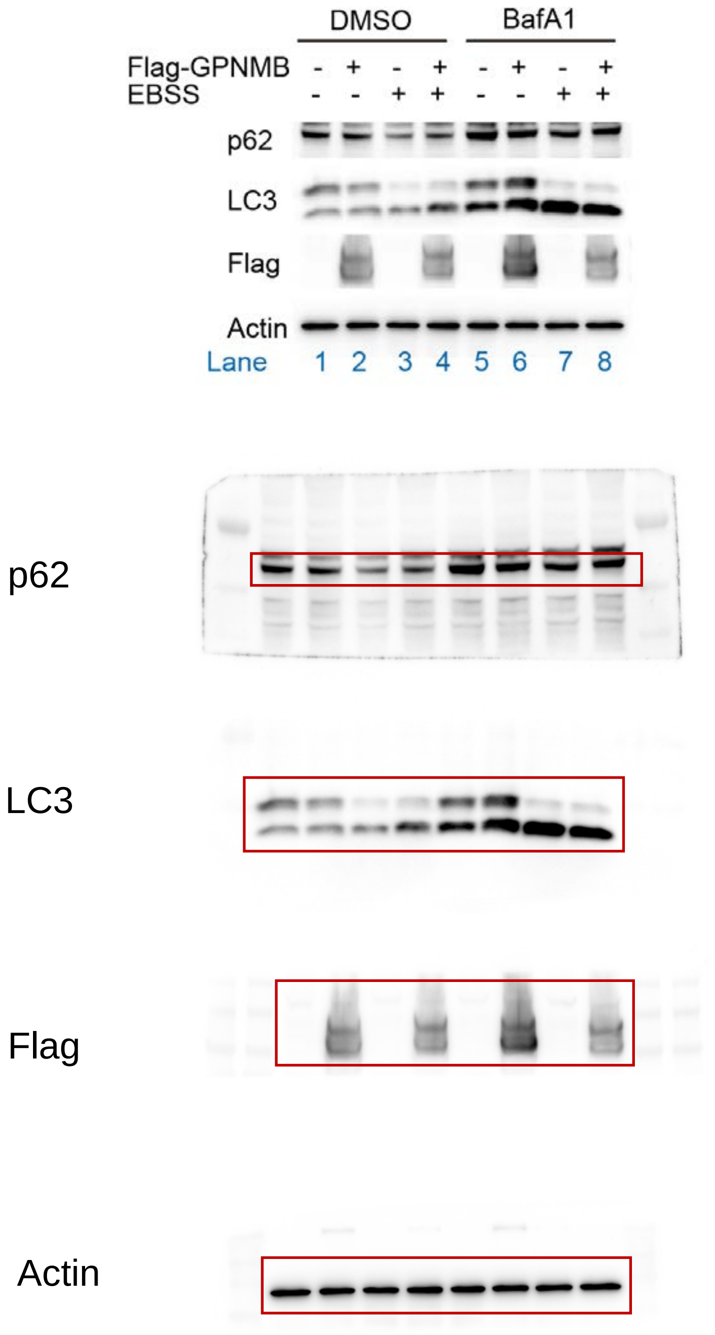
DMSO
BafA1
Flag-GPNMB - + - + - + - +
EBSS - - + + - - + +
p62
LC3
Flag
Actin
Lane 1 2 3 4 5 6 7 8
p62
LC3
Flag
Actin

Fig. 3L

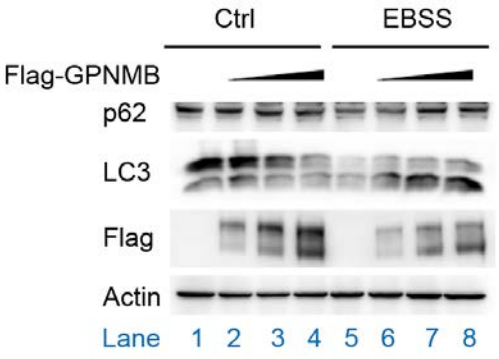
Ctrl
EBSS
Flag-GPNMB
p62
LC3
Flag
Actin
Lane 1 2 3 4 5 6 7 8

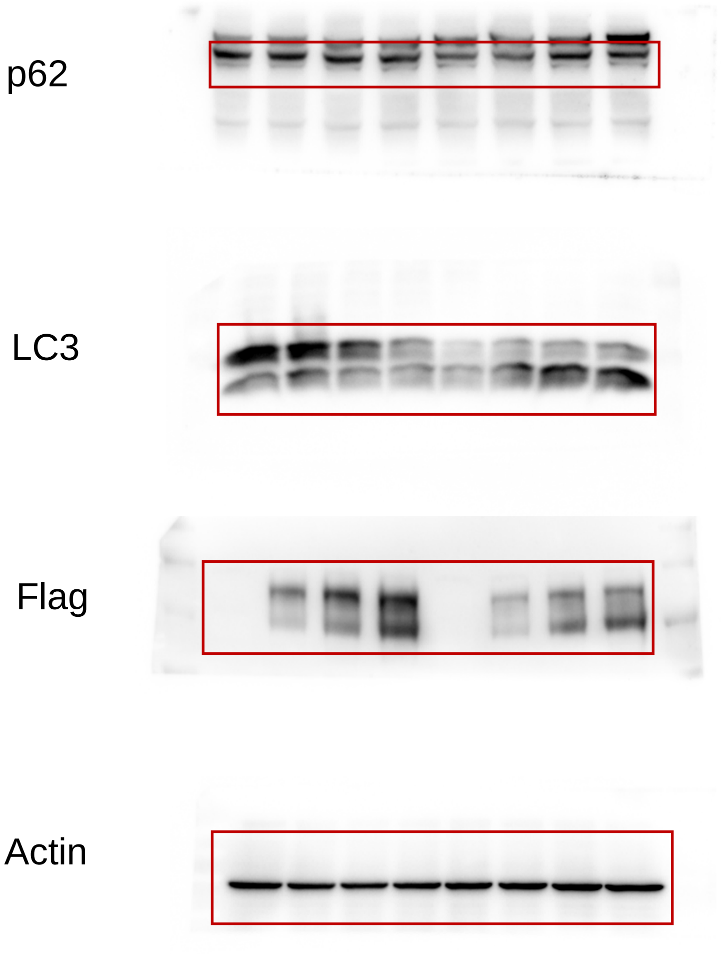
p62
LC3
Flag
Actin

Fig. 4A

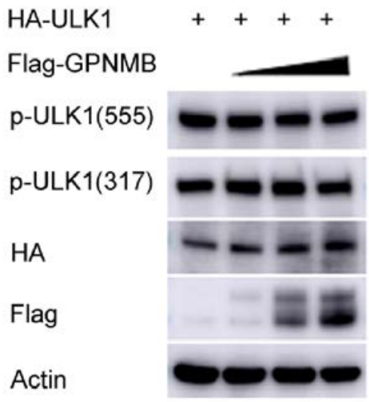

p-ULK1(555)

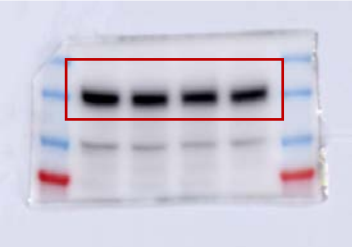

p-ULK1(317)

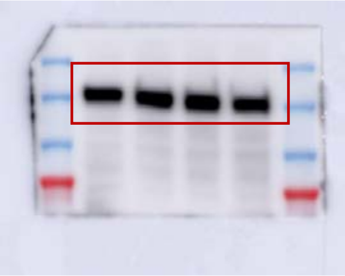

HA

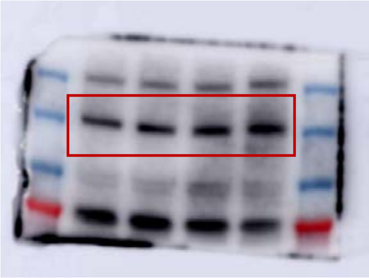

Flag

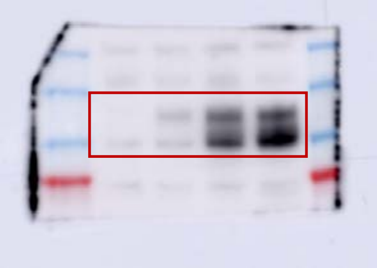

Actin

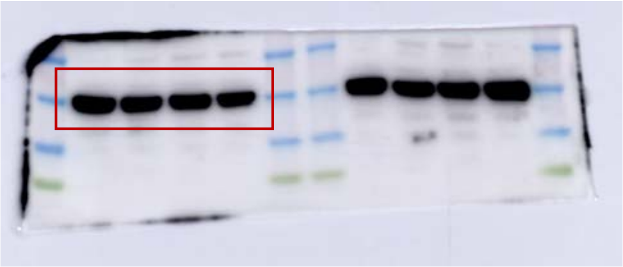

Fig. 4B

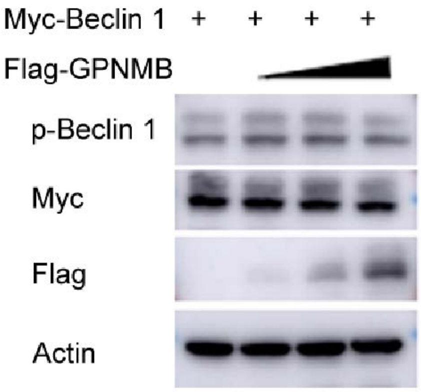

p-Beclin1

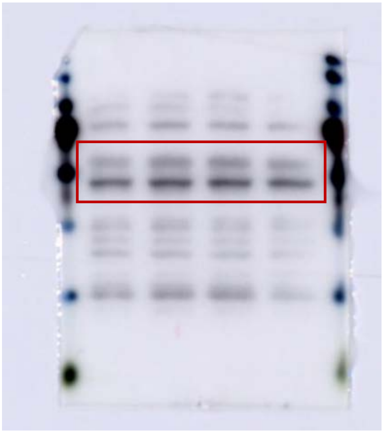

Myc

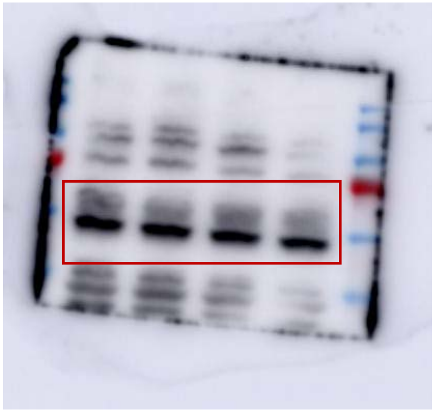

Flag

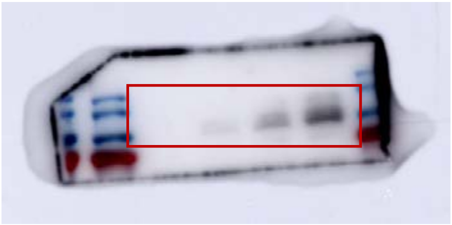

Actin

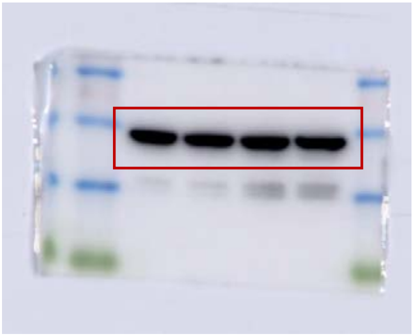

Fig. 4E

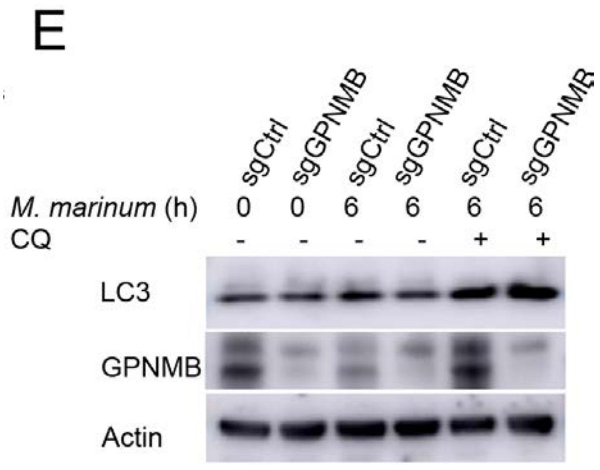

LC3

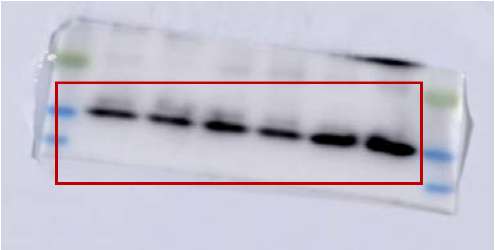

GPNMB

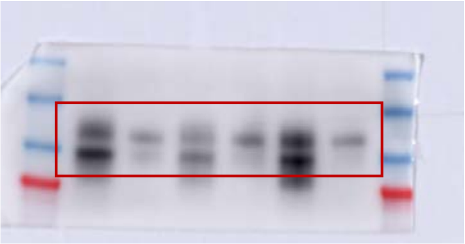

Actin

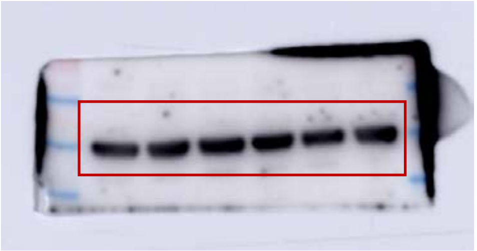

Fig. 5A

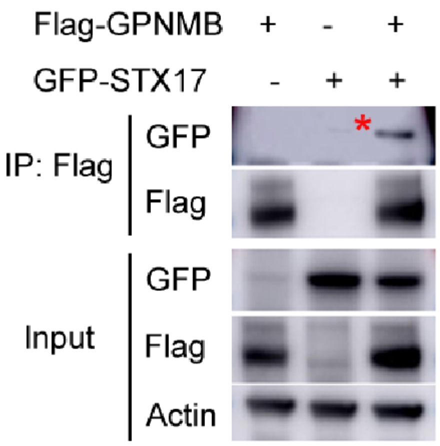
Flag-GPNMB + - +
GFP-STX17 - + +
IP: Flag
GFP
Flag
Input
GFP
Flag
Actin

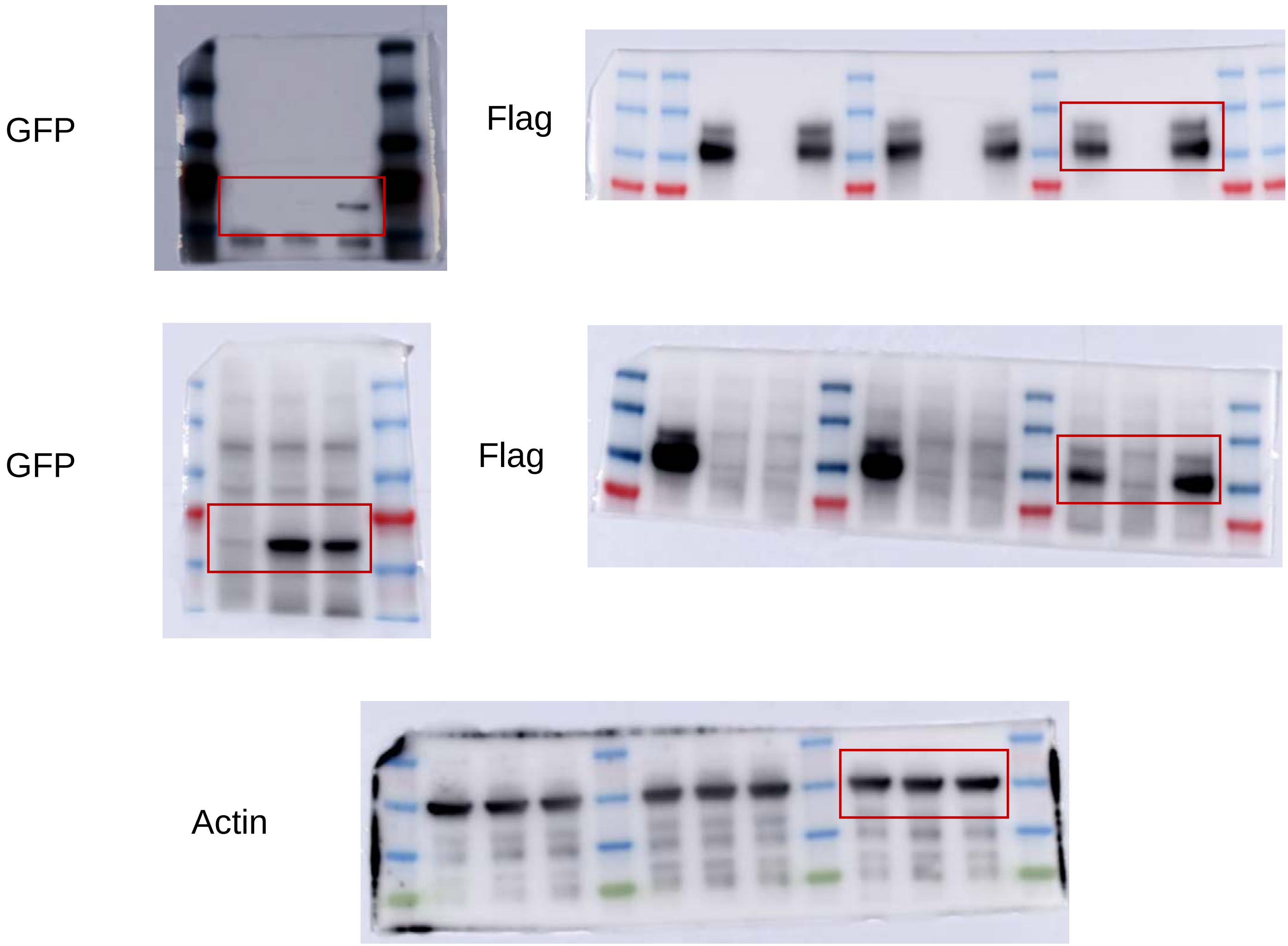
GFP
Flag
GFP
Flag
Actin

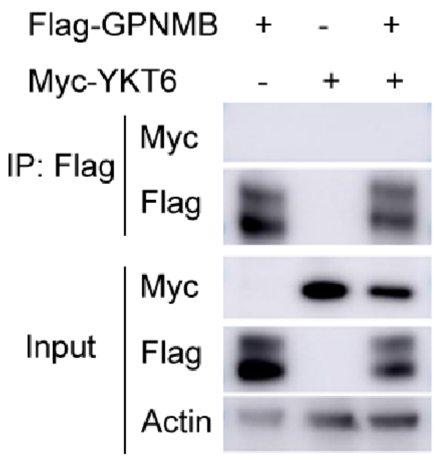

Myc

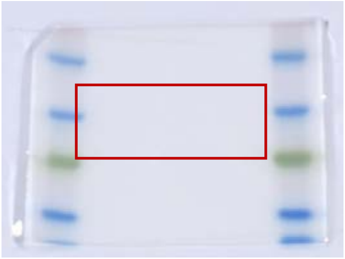

Flag

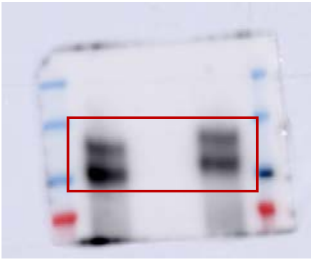

Myc

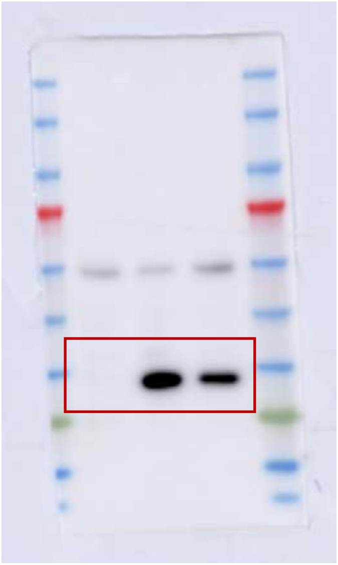

Flag

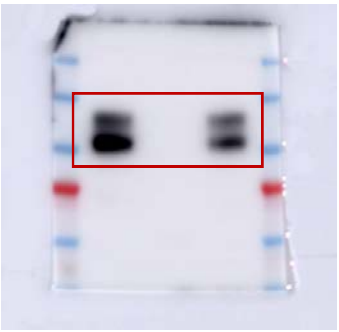

Actin

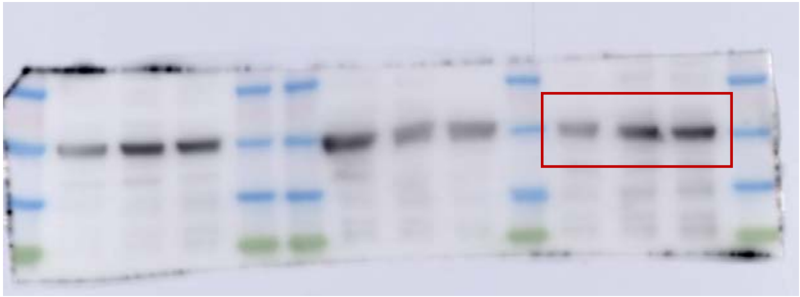

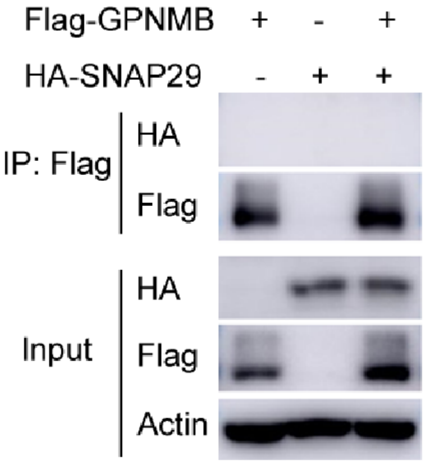

HA

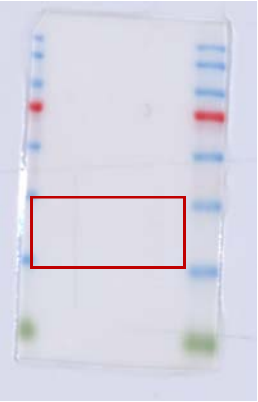

Flag

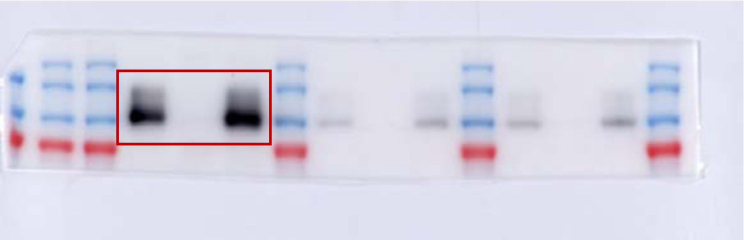

HA

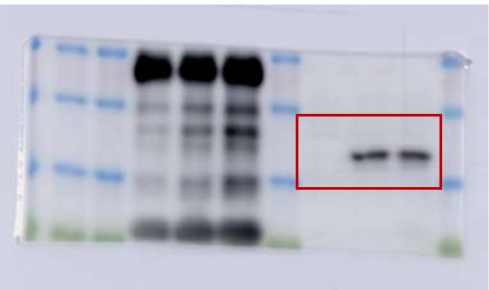

Flag

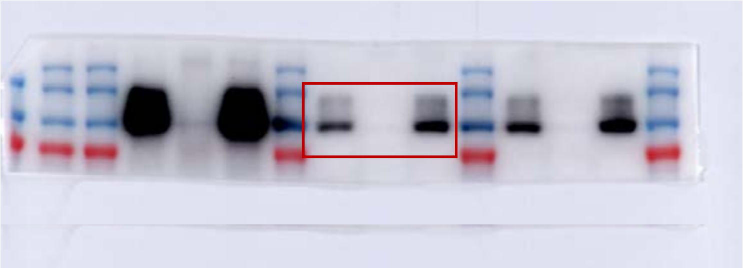

Actin

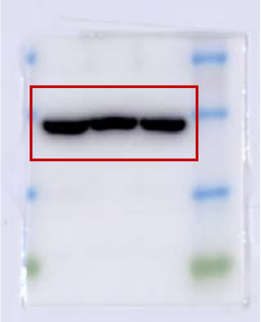

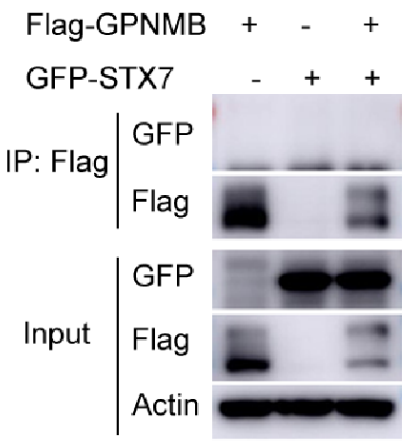
Flag-GPNMB + - +
GFP-STX7 - + +
IP: Flag
GFP
Flag
Input
GFP
Flag
Actin

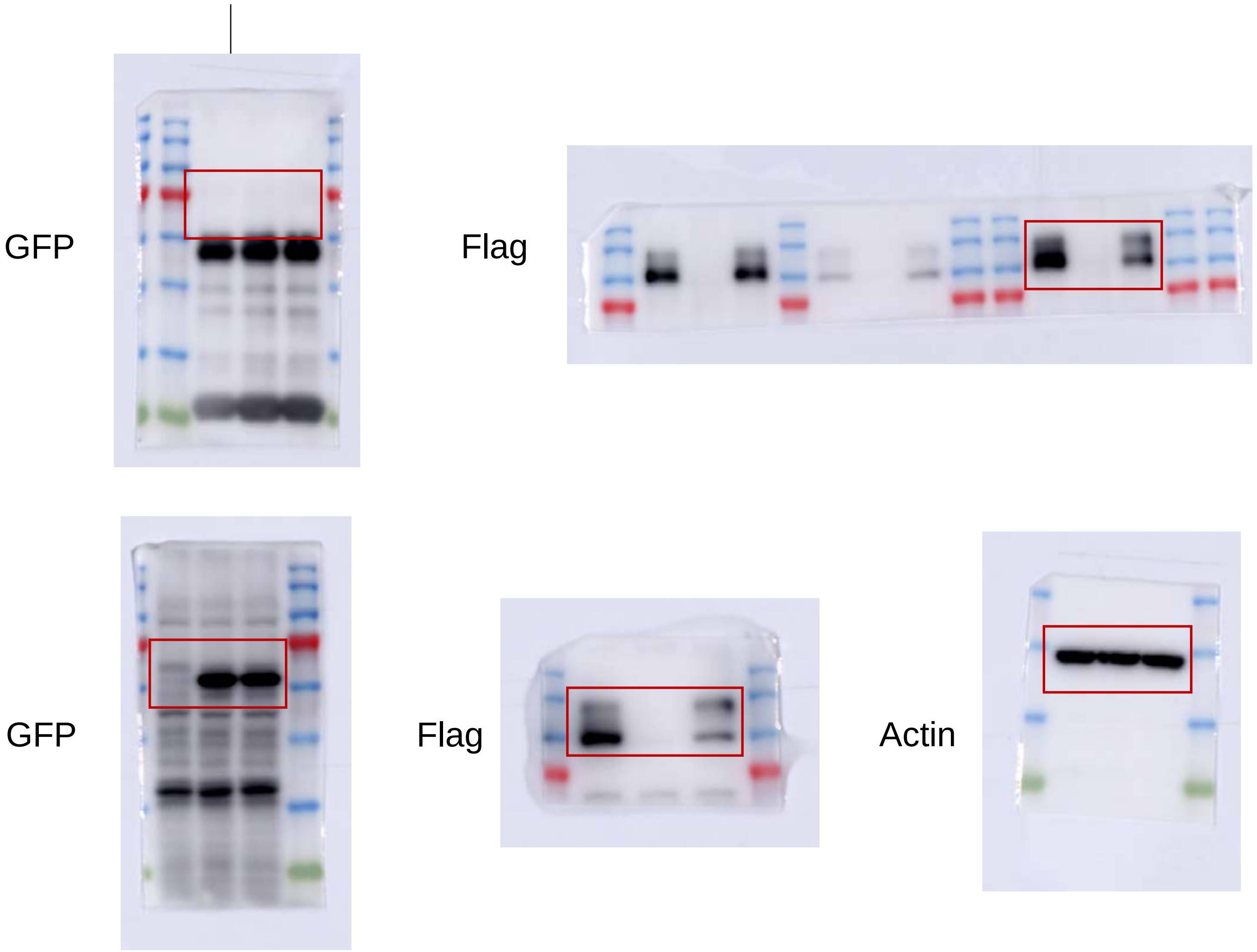
GFP
Flag
GFP
Flag
Actin

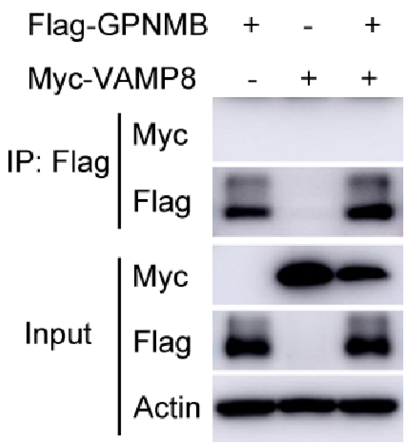

Myc

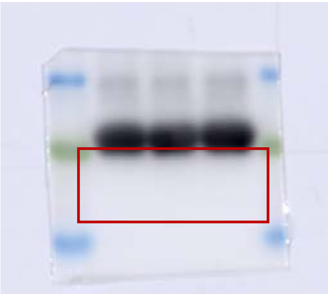

Flag

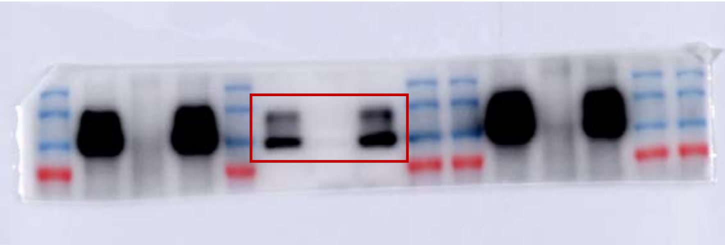

Myc

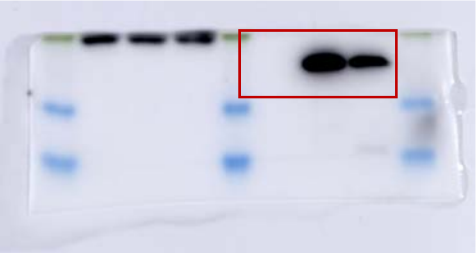

Flag

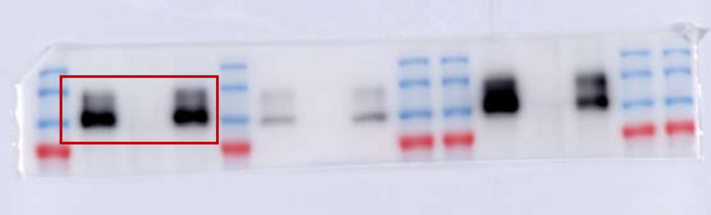

Actin

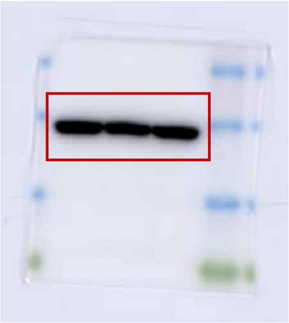

Fig. 5B

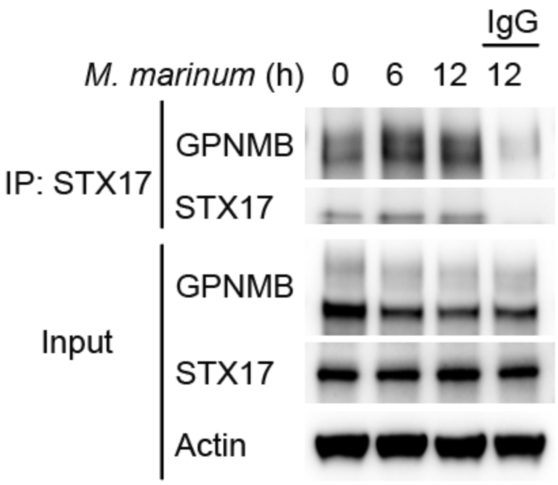
IgG
M. marinum (h) 0 6 12 12
IP: STX17
GPNMB
STX17
Input
GPNMB
STX17
Actin

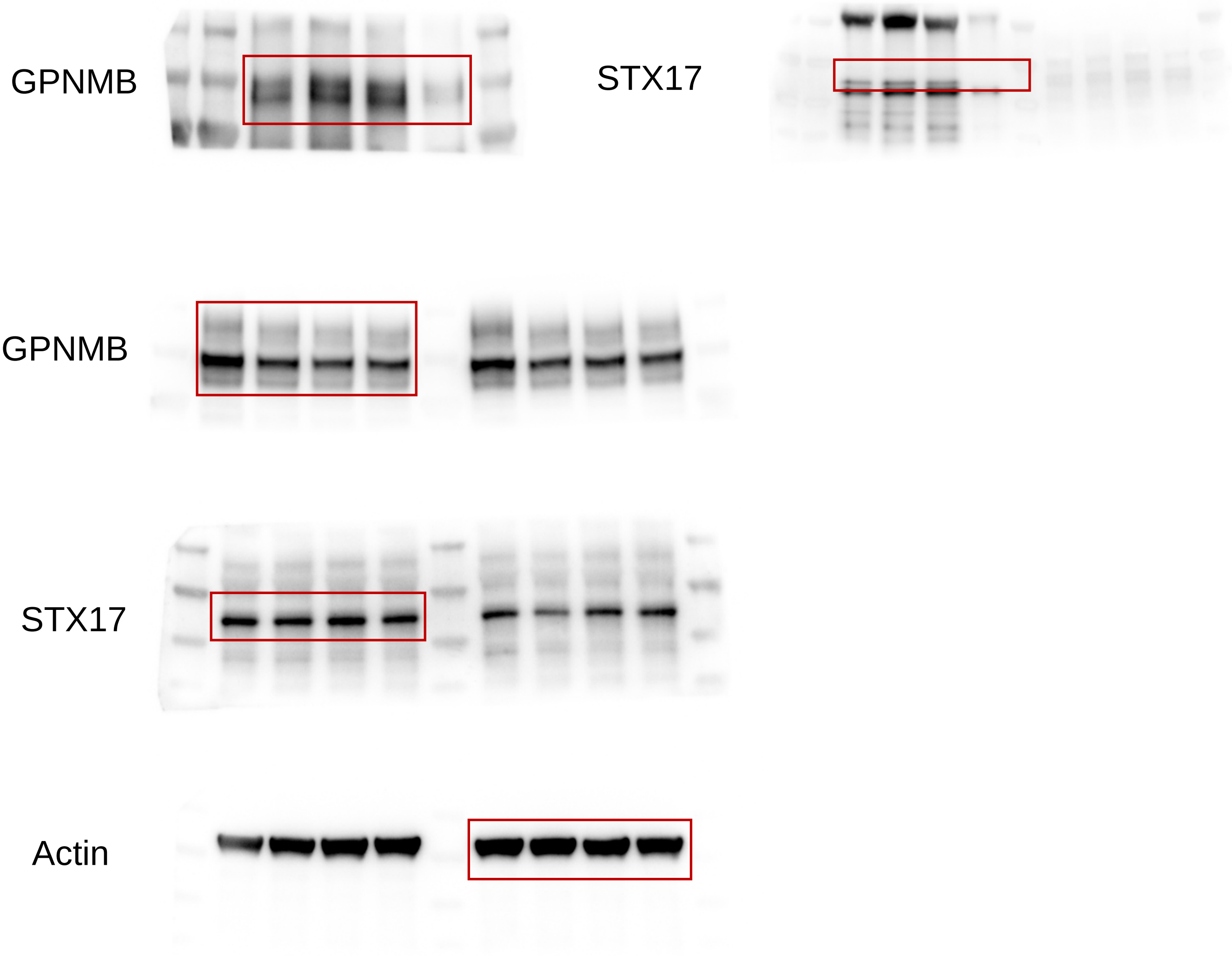
GPNMB
STX17
GPNMB
STX17
Actin

Fig. 5D

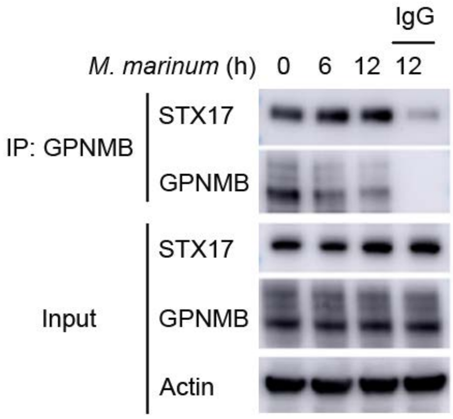

STX17

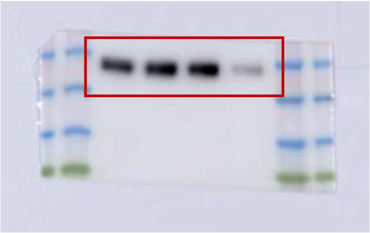

GPNMB

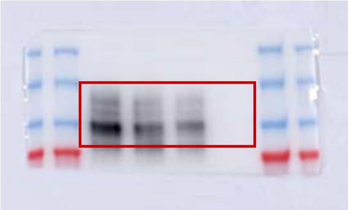

STX17

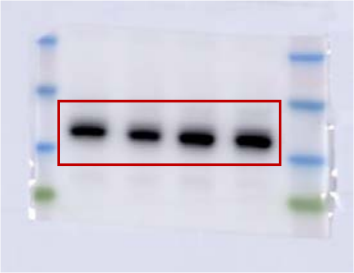

GPNMB

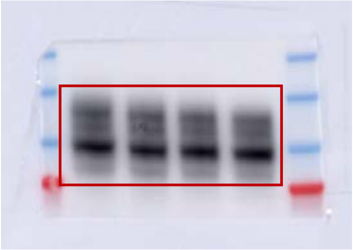

Actin

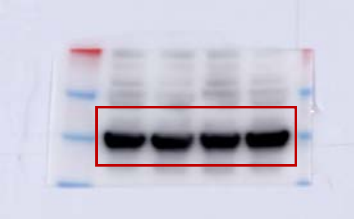

Fig. 5F

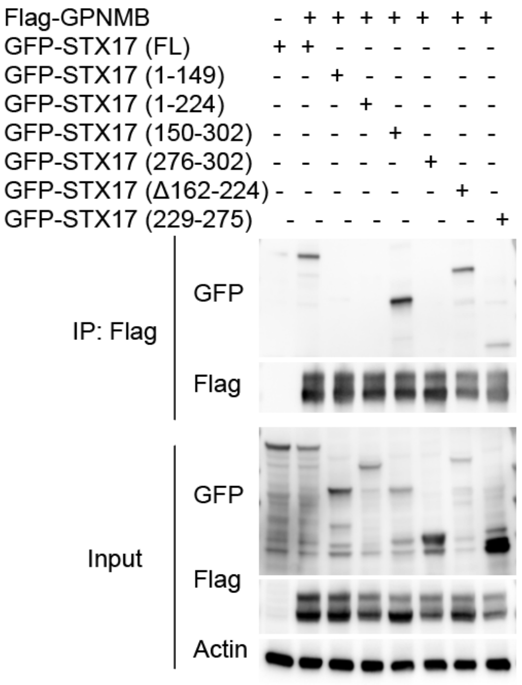
Flag-GPNMB - + + + + + + +
GFP-STX17 (FL) + + - - - - - -
GFP-STX17 (1-149) - - + - - - - -
GFP-STX17 (1-224) - - - + - - - -
GFP-STX17 (150-302) - - - - + - - -
GFP-STX17 (276-302) - - - - - + - -
GFP-STX17 (Δ162-224) - - - - - - + -
GFP-STX17 (229-275) - - - - - - - +
IP: Flag
GFP
Flag
Input
GFP
Flag
Actin

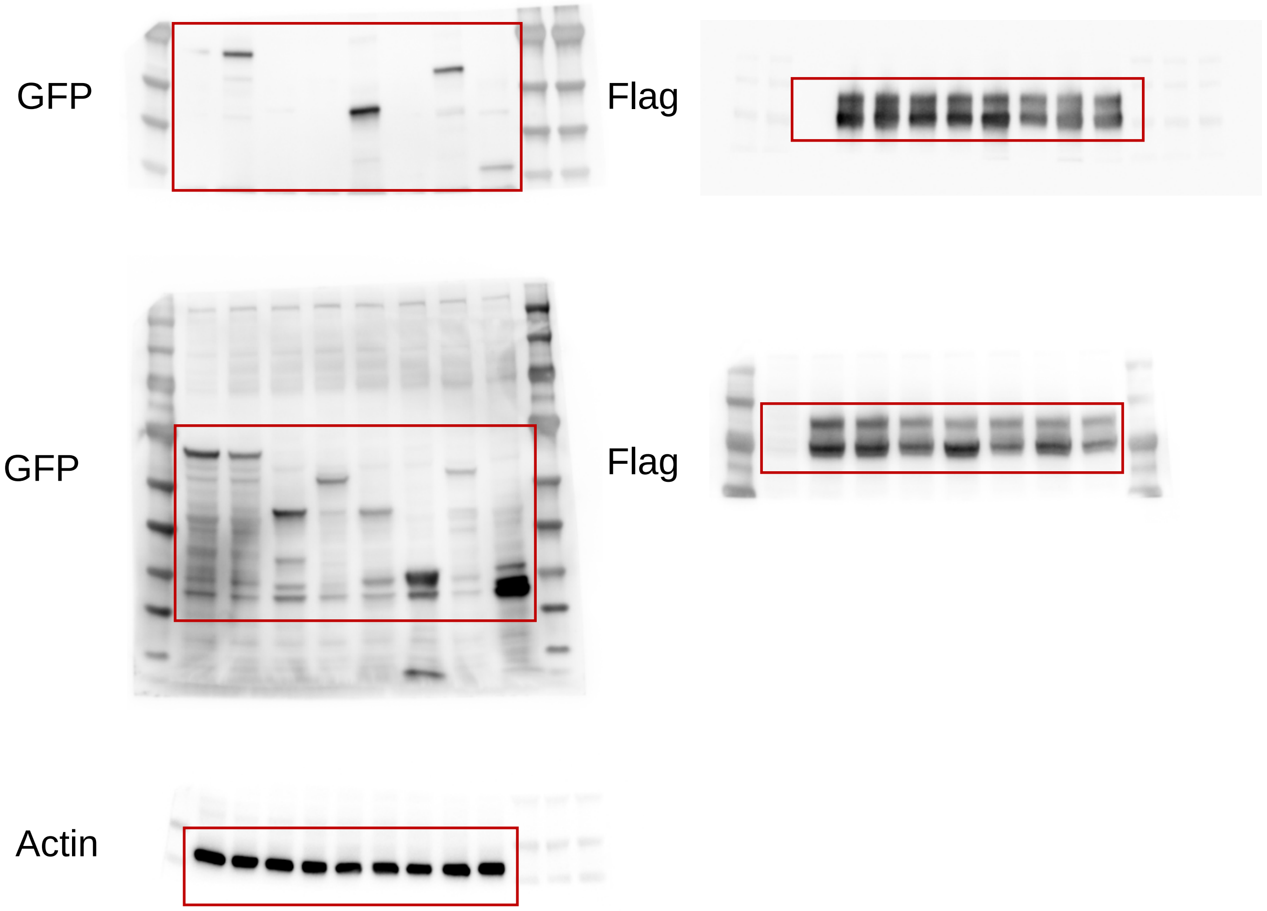
GFP
Flag
GFP
Flag
Actin

Fig. 6B

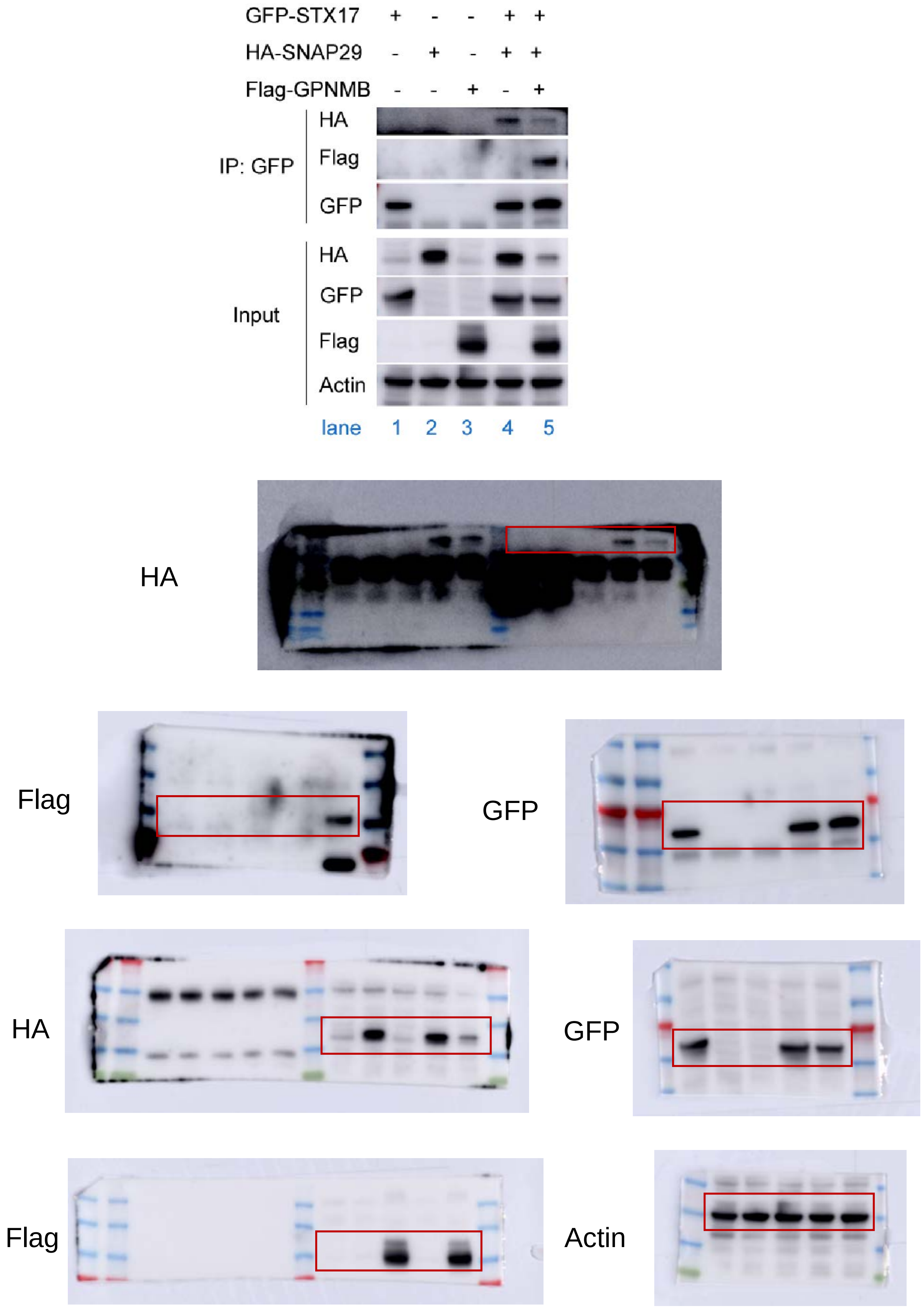
GFP-STX17 + - - + +
HA-SNAP29 - + - + +
Flag-GPNMB - - + - +
IP: GFP
HA
Flag
GFP
Input
HA
GFP
Flag
Actin
lane 1 2 3 4 5
HA
Flag
GFP
HA
GFP
Flag
Actin

Fig. 6D

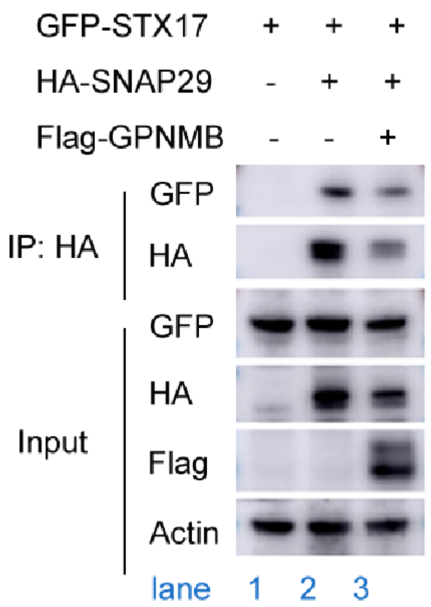
GFP-STX17 + + +
HA-SNAP29 - + +
Flag-GPNMB - - +
IP: HA
GFP
HA
Input
GFP
HA
Flag
Actin
lane 1 2 3

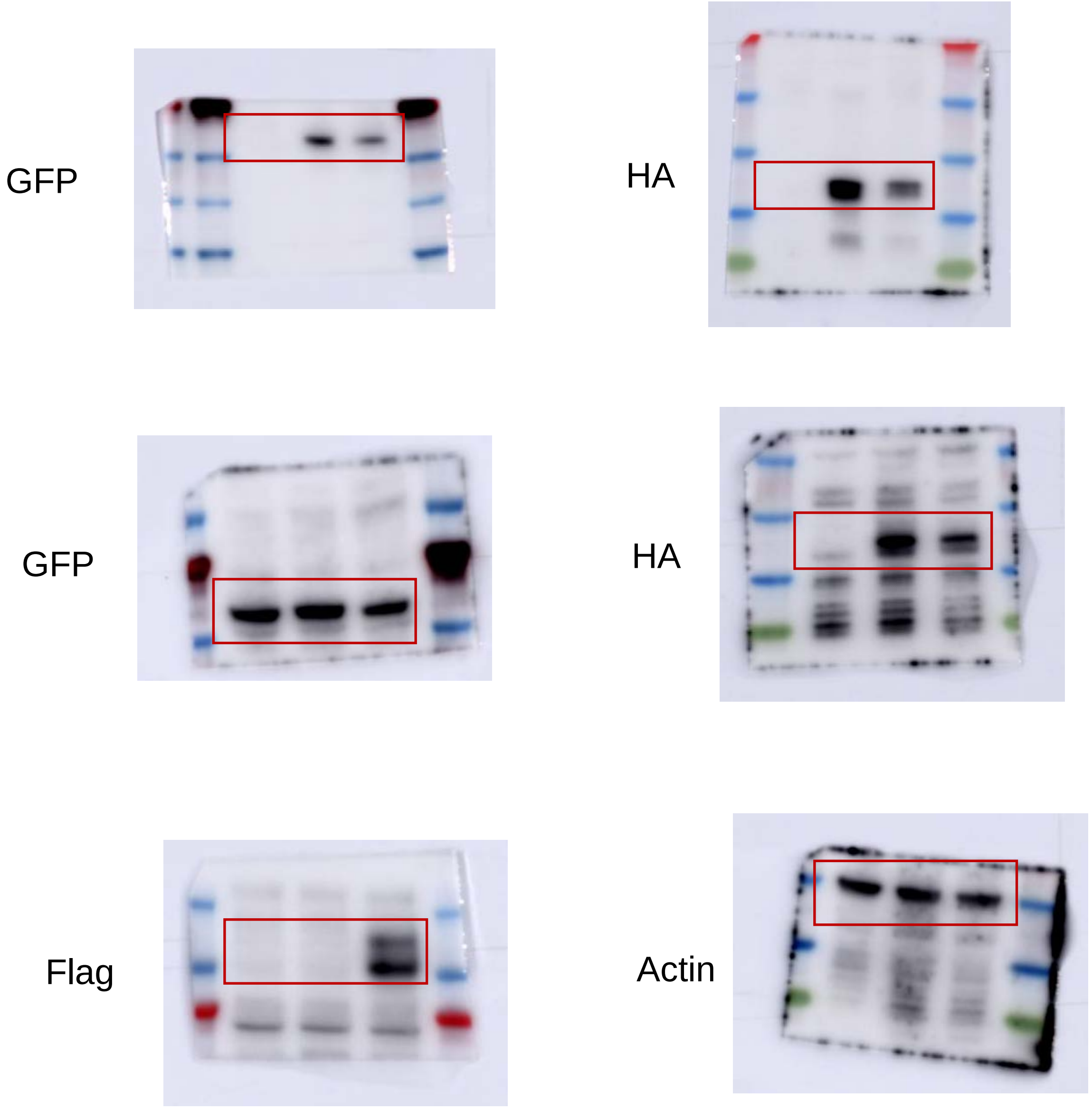
GFP
HA
GFP
HA
Flag
Actin

Fig.6F

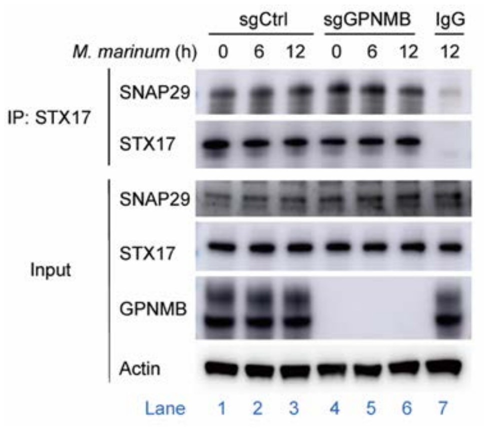

SNAP29

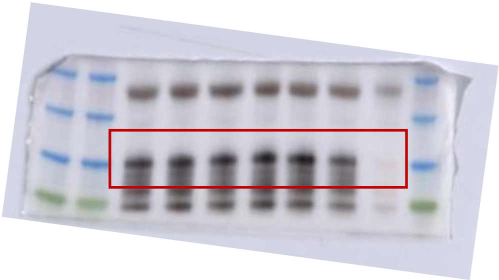

STX17

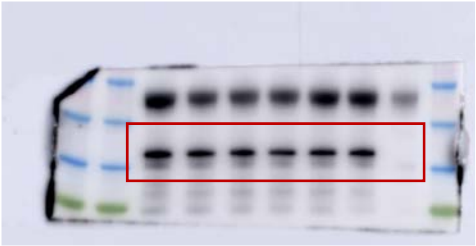

SNAP29

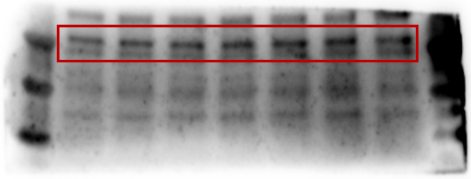

STX17

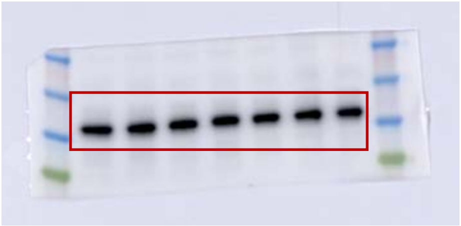

GPNMB

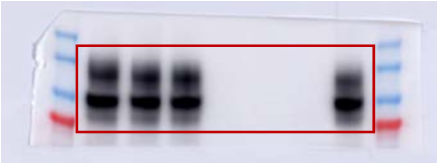

Actin

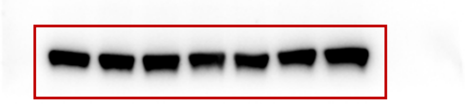

Fig 7A

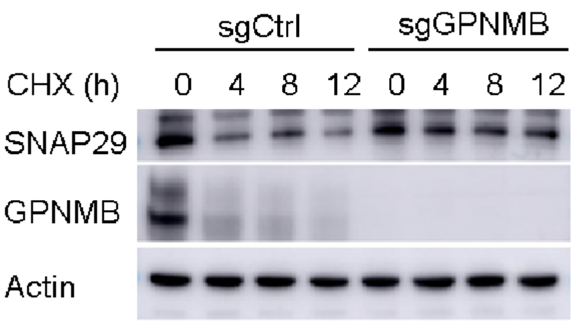

SNAP29

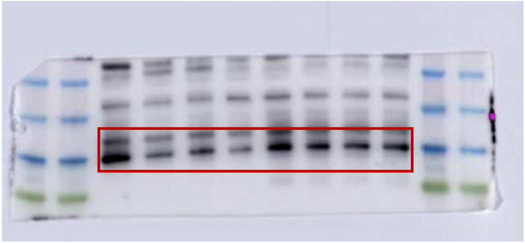

GPNMB

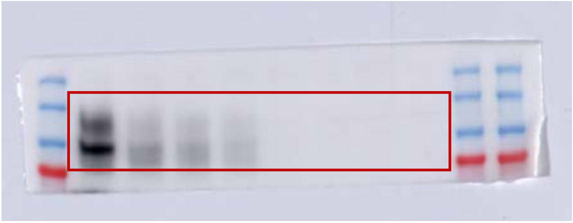

Actin

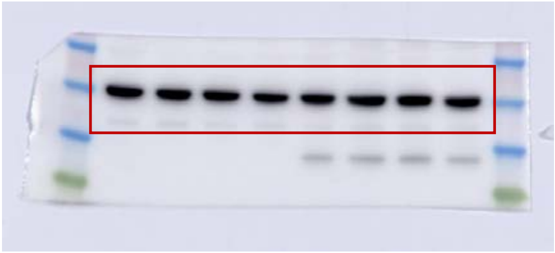

Fig 7B

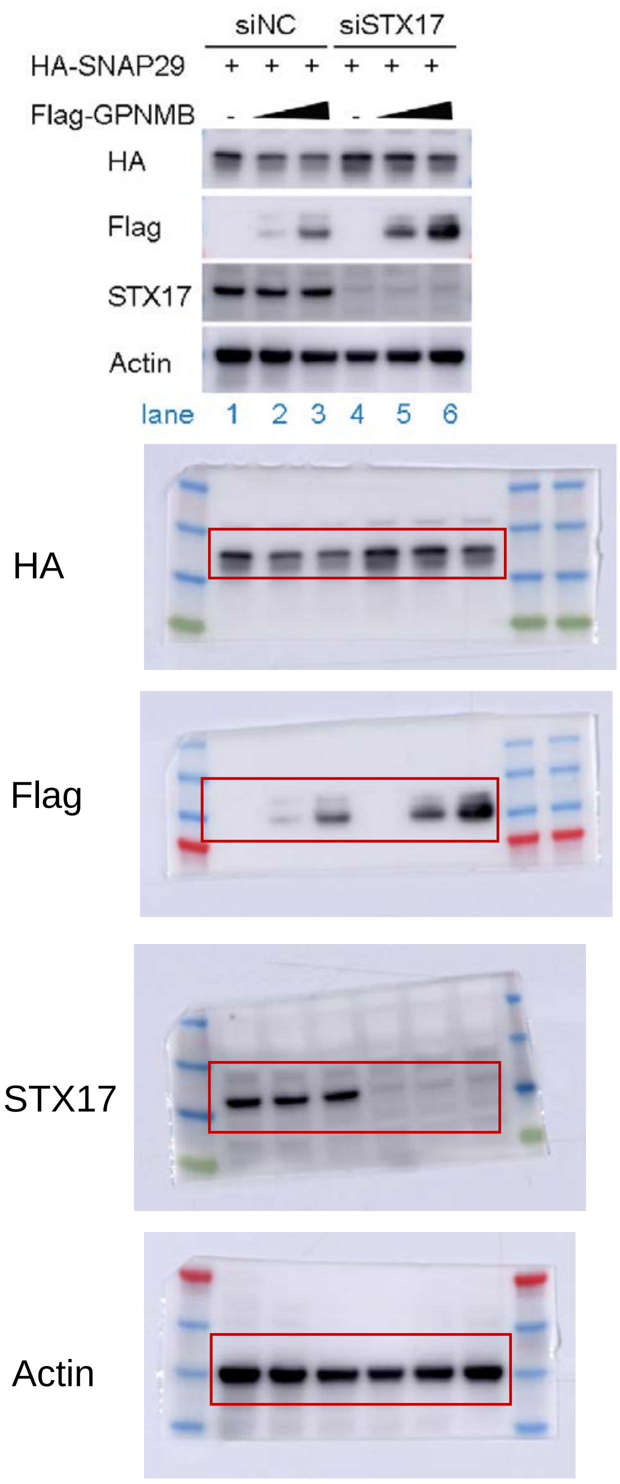
siNC
siSTX17
HA-SNAP29
+ + + + + +
Flag-GPNMB
- -
HA
Flag
STX17
Actin
lane 1 2 3 4 5 6
HA
Flag
STX17
Actin

Fig 7D

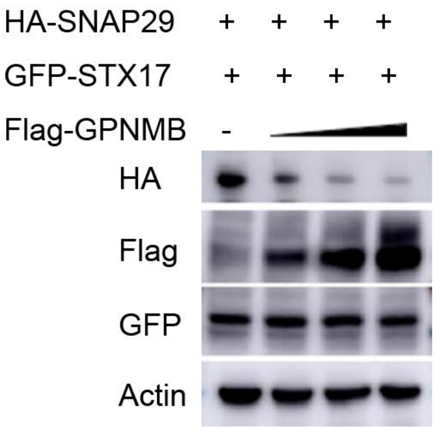
HA-SNAP29 + + + +
GFP-STX17 + + + +
Flag-GPNMB -
HA
Flag
GFP
Actin

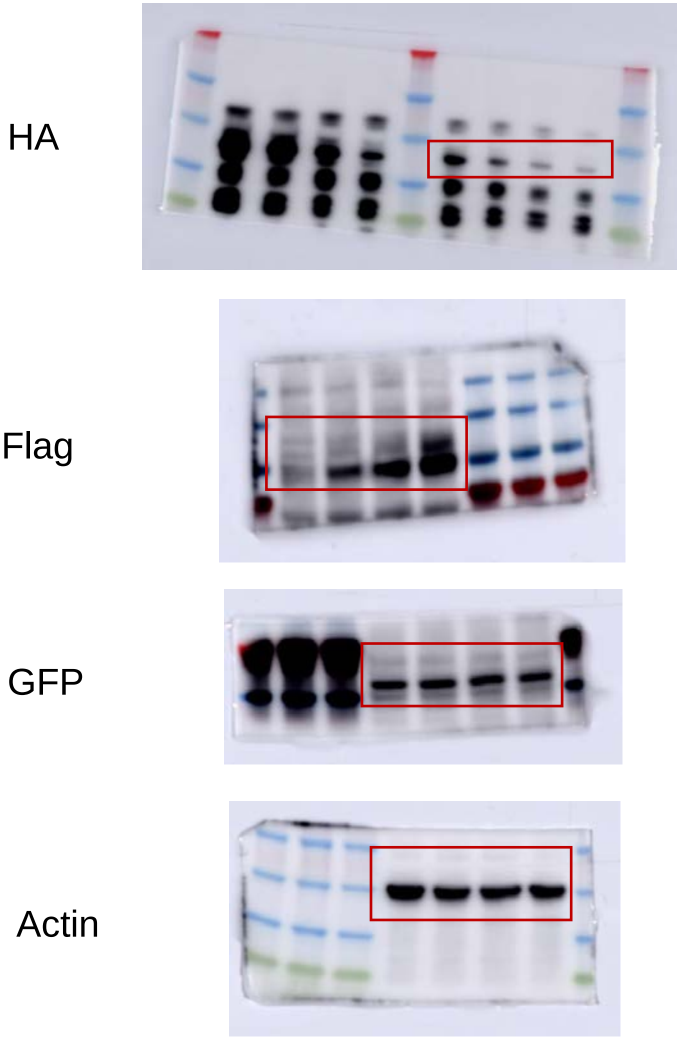
HA
Flag
GFP
Actin

Fig. 7G

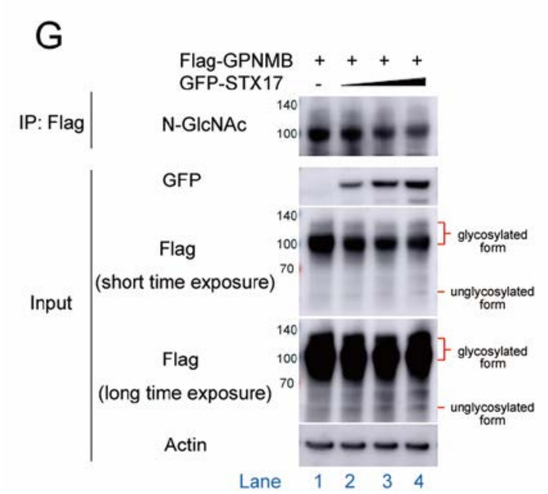

N-GlcNAc

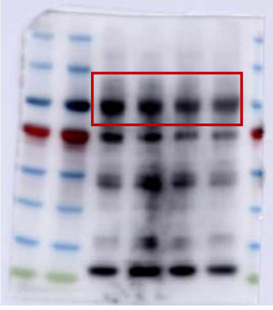

GFP

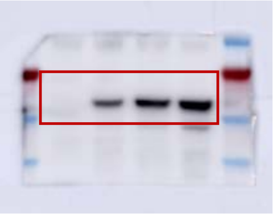

Flag

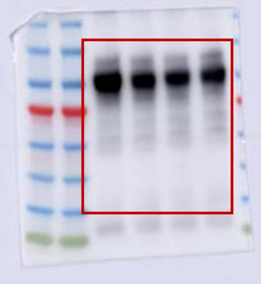

Flag

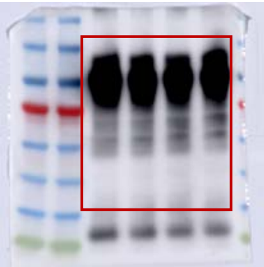

Actin

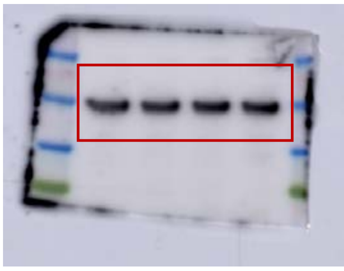

Fig. 7I

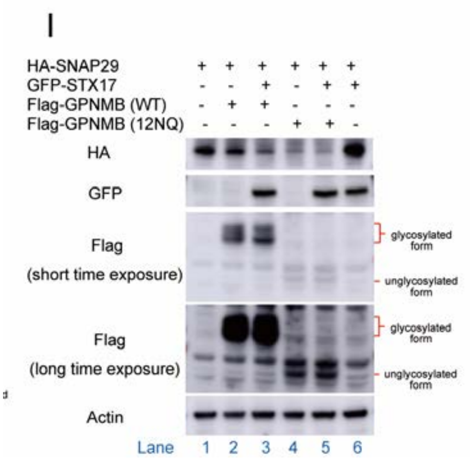

HA

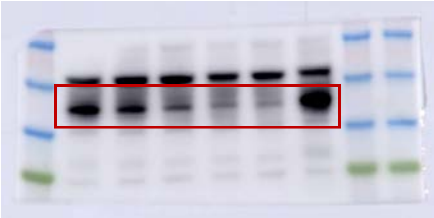

GFP

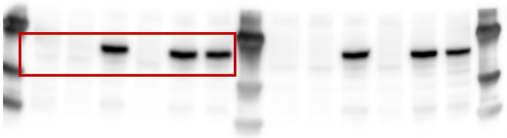

Flag

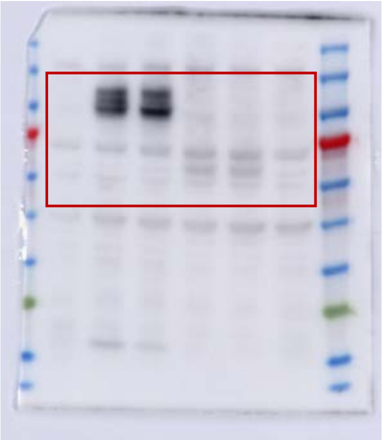

Flag

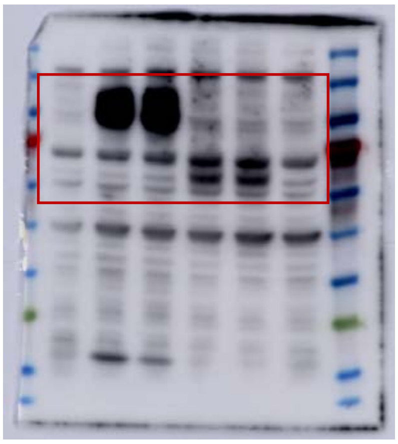

Actin

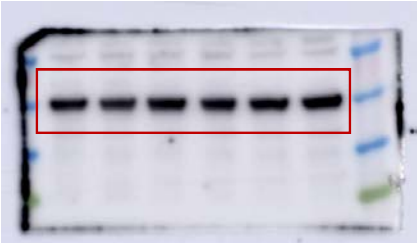

Fig. 7J

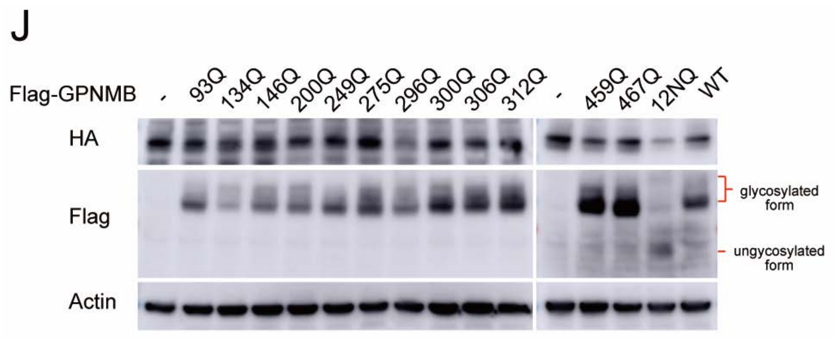

HA

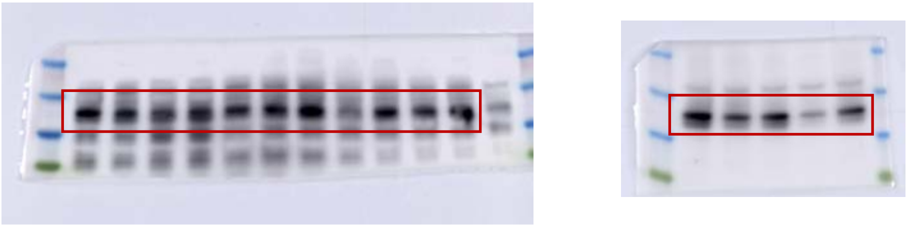

Flag

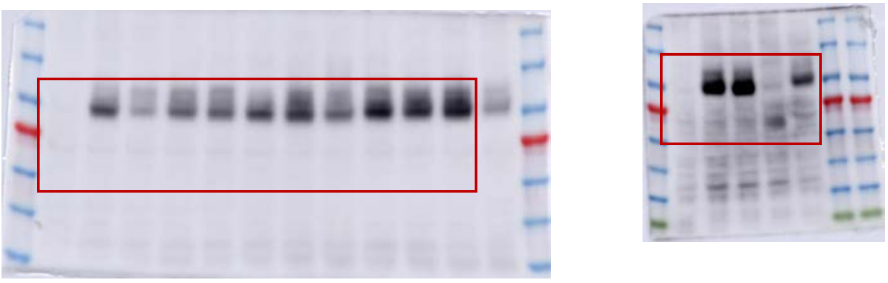

Actin

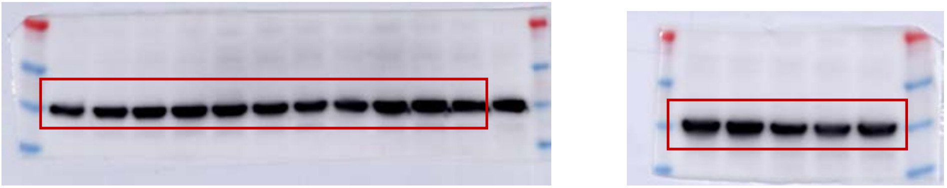

Fig. 7L

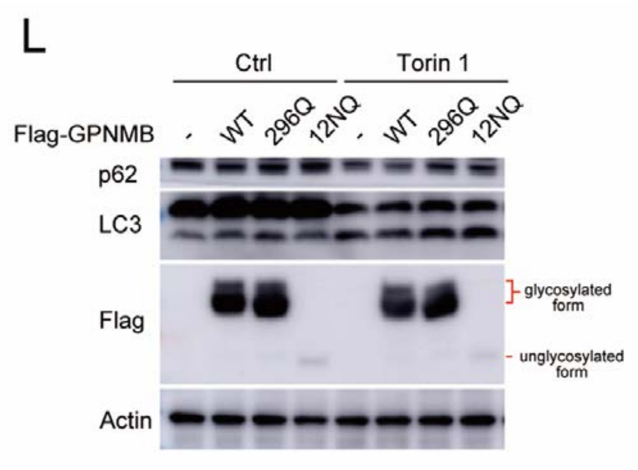

p62

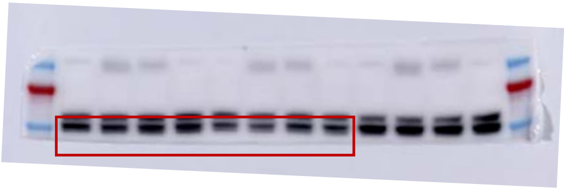

LC3

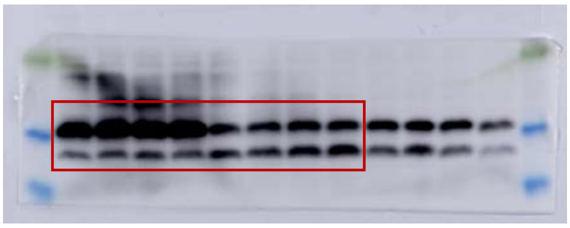

Flag

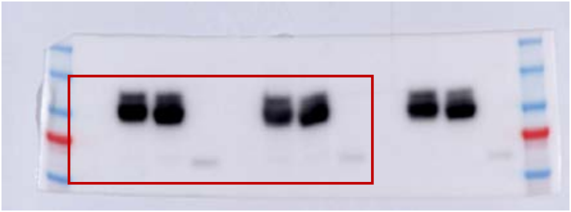

Actin

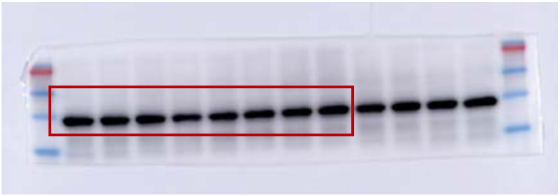

Supplementary Fig S1A

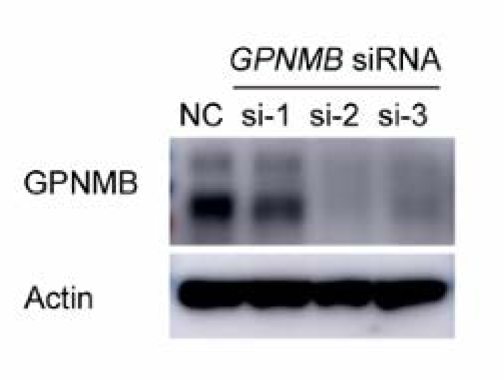

GPNMB

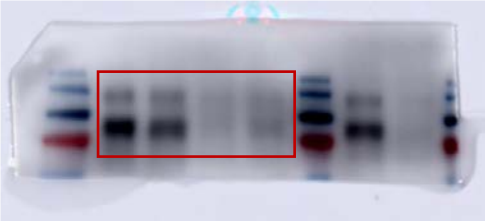

Actin

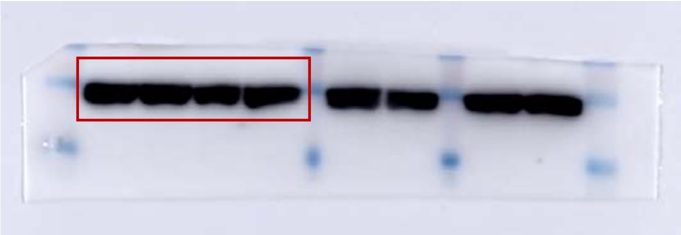

Supplementary Fig S1C

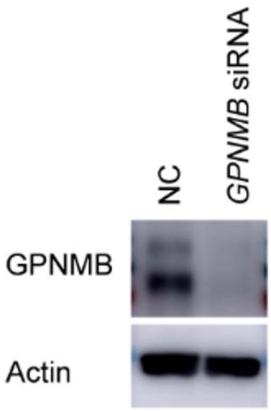

GPNMB

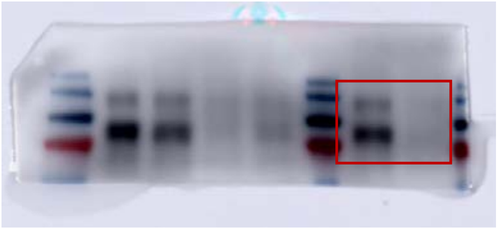

Actin

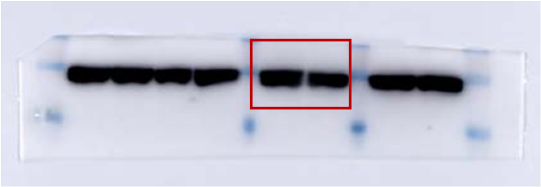

Supplementary Fig S7A

A

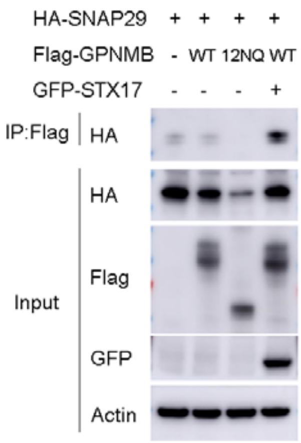

HA

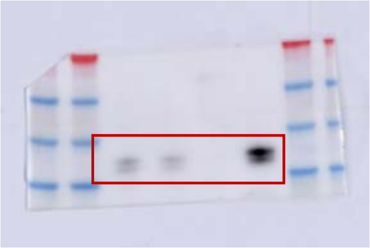

HA

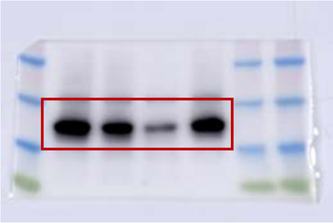

Flag

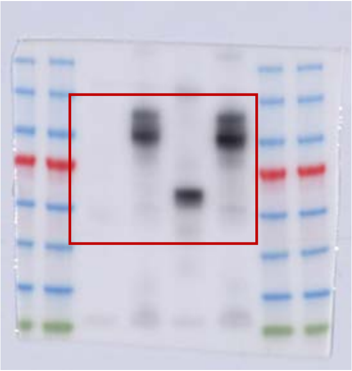

GFP

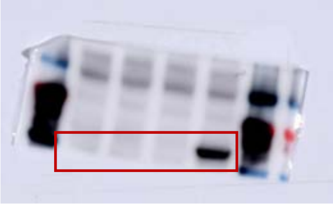

Actin

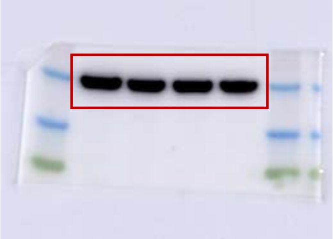

Supplementary Fig S7B

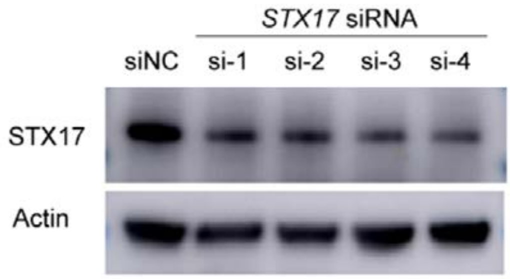

STX17

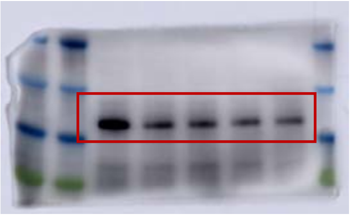

Actin

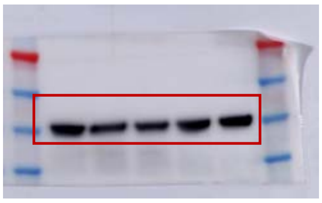

Supplementary Fig S8

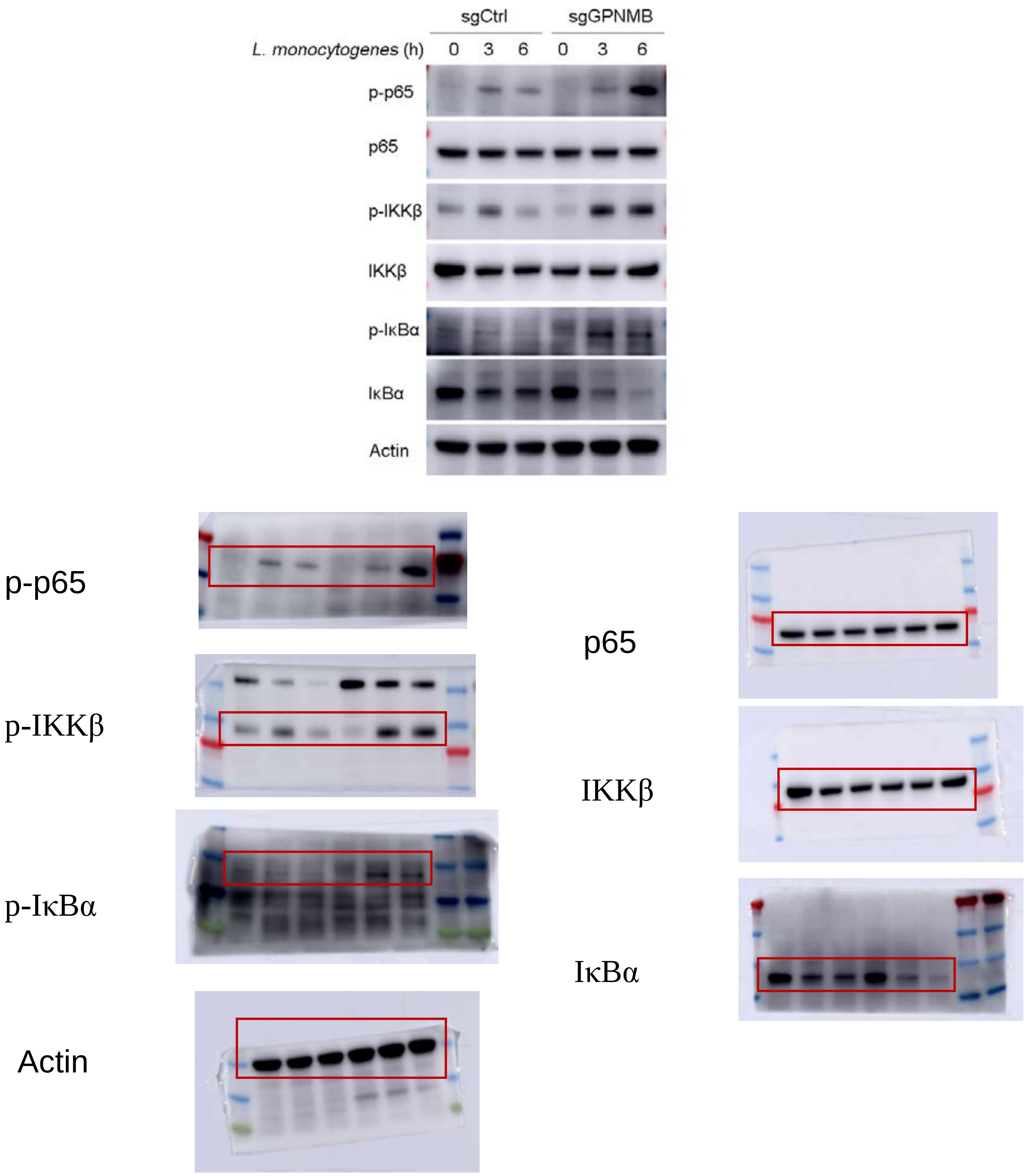

Supplement: Supplementary file 2 — Supplementary-original images of gels [file 41423_2025_1272_MOESM2_ESM.pdf]
